# Supplementary material for: Revealing hidden patterns in deep neural network feature space continuum via manifold learning
Source: Nat Commun. 2023 Dec 21;14:8506. doi: 10.1038/s41467-023-43958-w (PMC10739971; doi:10.1038/s41467-023-43958-w)
Supplement: Supplementary file 1 — Supplementary Information [file 41467_2023_43958_MOESM1_ESM.pdf]

Revealing Hidden Patterns in Deep Neural Network Feature Space Continuum via Manifold Learning

Md Tauhidul Islam et al.

Contents

1 List of notations 4

2 Choice of distributions in Bayesian dimensionality reduction 4

3 How MDA preserves global and local manifold structure? 5

4 Relationship between PCA and MDS 7

5 Distribution of distance values in different tasks 7

6 Visualization of features for a deep regression task 11

7 Visualization of features at intermediate epochs 22

8 Quantification of the manifold structure 26

9 Neural collapse in regression tasks 29

10 Effect of different layers on DNN feature space 32

11 Analysis of DNN features for extrapolation tasks 35

12 Effect of different hyperparameters on MDA visualizations 39

13 Effect of noise on DNN feature space 43

14 Supplementary References 48

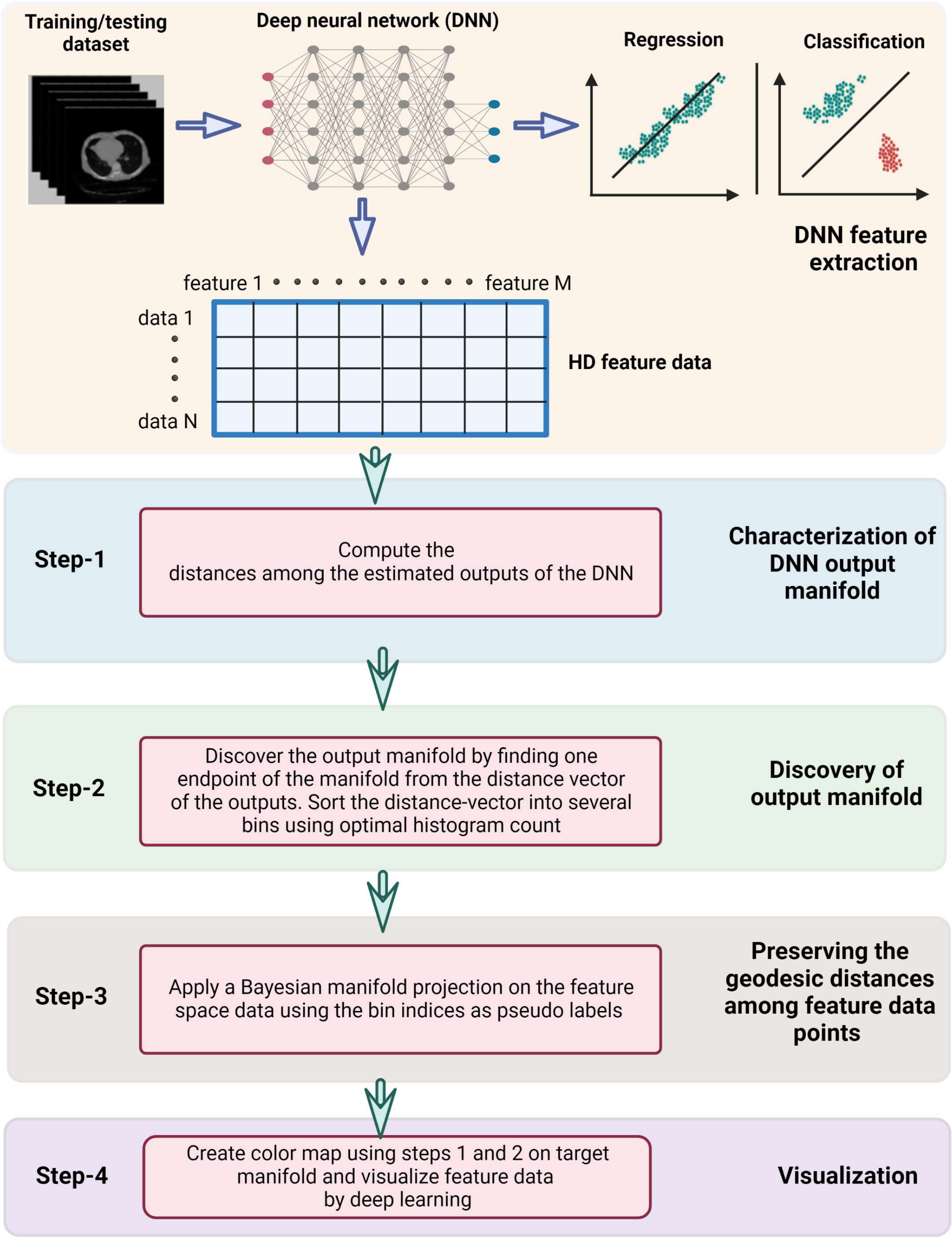

Fig. S1. Step by step work flow of MDA (manifold discovery analysis). i) The distances among the estimated outputs of the DNN are computed. The starting point of the manifold is found by finding the point with the maximum distance value. The distance of all the outputs from the starting point results in a distance vector of length equal to the number of data points. ii) The distance values are sorted into K number of bins. MDA uses an automatic binning algorithm that returns K bins with a uniform width, chosen to cover the range of elements in the distance vector and reveal the underlying shape of the distribution. The discretized distance vector works as the data labels in the next step of MDA. iii) A conditional Bayesian approach is applied on the training/test data with discretized distance values as the labels. iv) A deep learning-based dimensionality reduction technique is applied to the Bayesian components to obtain the MDA results. Color map for the visualization is created using steps 1 and 2 from the actual labels. This figure was created with BioRender.com.

1. List of notations

Table S1. List of notation

n	Number of training instances
K	Number of classes
D	Dimensionality of input space
R	Dimensionality of projected subspace
$\mathbf{X}$	$D \times n$ matrix of input data
$\mathbf{Q}$	$D \times R$ matrix of projection variables
Φ	$D \times R$ matrix of precision priors over projection variables
$\mathbf{Z}$	$R \times n$ matrix of projected variables
$\mathbf{W}$	$R \times K$ matrix of weight parameters
Ψ	$R \times K$ matrix of precision priors over weight parameters
b	$K \times 1$ vector of bias parameters
λ	$K \times 1$ vector of precision priors over bias parameters
$\mathbf{T}$	$n \times K$ matrix of score variables
$\boldsymbol{y}$	$n \times 1$ vector of associated target values from $\{1, \ldots, K\}$

Table S2. Probability distribution function of different variables in Bayesian projection in MDA

ϕ_s^f	$\sim \mathcal{G}\left(\phi_s^f; \alpha_\phi, \beta_\phi\right)$	$\forall(f,s)$
$q_s^f \mid \phi_s^f$	$\sim \mathcal{N}\left(q_s^f; 0, \left(\phi_s^f\right)^{-1}\right)$	$\forall(f,s)$
$z_i^s \mid \boldsymbol{q}_s, \boldsymbol{x}_i$	$\sim \mathcal{N}\left(z_i^s; \boldsymbol{q}_s^\top \boldsymbol{x}_i, 1\right)$	$\forall(s,i)$
λ_c	$\sim \mathcal{G}\left(\lambda_c; \alpha_\lambda, \beta_\lambda\right)$	$\forall c$
$b_c \mid \lambda_c$	$\sim \mathcal{N}\left(b_c; 0, \lambda_c^{-1}\right)$	$\forall c$
ψ_c^s	$\sim \mathcal{G}\left(\psi_c^s; \alpha_\psi, \beta_\psi\right)$	$\forall(s,c)$
$w_c^s \mid \psi_c^s$	$\sim \mathcal{N}\left(w_c^s; 0, (\psi_c^s)^{-1}\right)$	$\forall(s,c)$
$t_i^c \mid b_c, w_c, z_i$	$\sim \mathcal{N}\left(t_i^c; w_c^\top z_i + b_c, 1\right)$	$\forall(c,i)$
$y_i \mid t_i$	$\sim \prod_{c \neq y_i} \delta\left(t_i^{y_i} > t_i^c\right)$	$\forall i$

2. Choice of distributions in Bayesian dimensionality reduction

The choice of distributions (Gamma and normal distribution) in the Bayesian dimensionality reduction are related to the notion of conjugate priors in Bayesian statistics (1). Specifically, such distributions offer a way to ensure that posterior distributions belong to the same parametric family as the prior distributions, which can make calculations more tractable. Here are some reasons for the use of conjugate priors:

Analytical tractability: Conjugate priors lead to closed-form solutions for the posterior distribution. This can simplify calculations and make it easier to derive analytical expressions for posterior means, variances, and other summary statistics. This is especially useful when performing Bayesian updates sequentially, as in the case of updating beliefs as new data is collected.

Simplified updating: When we have a conjugate prior, the updated posterior distribution can be expressed in terms of known hyperparameters. This allows us to easily update our beliefs with new data by simply adjusting these hyperparameters. This is in contrast to the non-conjugate priors, where numerical methods like Markov Chain Monte Carlo (MCMC) may be required for estimation.

More specifically, in the Bayesian dimensionality reduction method, the choice of Gamma and Normal distributions allow one to compute a closed form solution for the posterior distribution $q(\mathbf{Q})$ that approximates $p(\mathbf{Q} \mid \mathbf{X}, \mathbf{y})$, which in turn lends itself to a simple closed form expression for the distribution of the projected vector $p\left(\mathbf{u}^* \mid \mathbf{x}^*, \mathbf{Q}, \mathbf{X}, \hat{\mathbf{y}}\right)$.

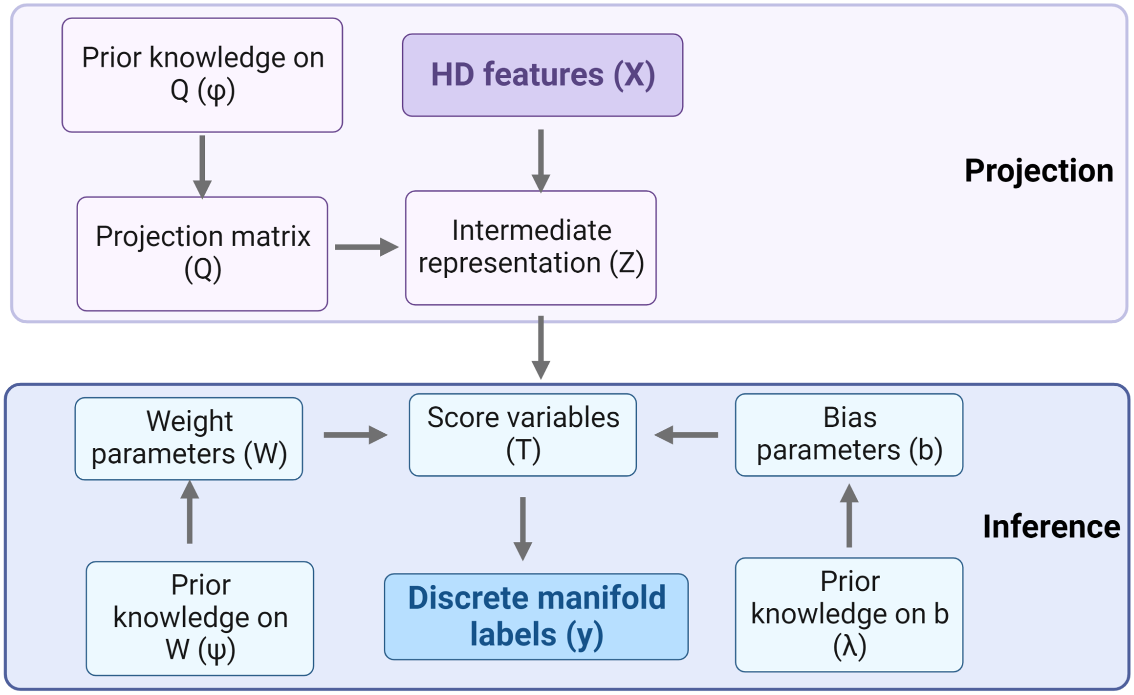

Fig. S2. Bayesian projection in MDA. Projection and inference are jointly performed to obtain an intermediate representation of the HD feature data.

3. How MDA preserves global and local manifold structure?

Preserving Global Structure. Preserving global structure means retaining the overall patterns, trends, and variations in the dataset. In mathematical terms, this often translates to preserving the high-variance directions in the data. For instance, principal component analysis (PCA) retains the global structure of data via finding orthogonal axes that maximize variation of data. Please note that for mean subtracted data (each column of $\boldsymbol{X}$ has zero mean), PCA provides the same result as classical multi-dimensional scaling and preserves the Euclidean distance of the data points (proof is added as supplementary section 4). Thus, for the mean subtracted data, 'maximizing the data variation in the projection domain' translates to 'preserving global Euclidean distance'. MDA is based on a Bayesian dimensionality reduction method that samples the projection matrix $\mathbf{Q}$ from a Gaussian distribution as follows (see Eq. 16 in the main manuscript)

$$q(\mathbf{Q}) = \prod_{s=1}^{R} \mathcal{N}\left(\boldsymbol{q}_s; \Sigma\left(\boldsymbol{q}_s\right) \mathbf{X}\widetilde{\boldsymbol{z}}^s, \left(\operatorname{diag}\left(\widetilde{\boldsymbol{\phi}_s}\right) + \mathbf{X}\mathbf{X}^\top\right)^{-1}\right). \tag{1}$$

As we prove below, very similar to PCA, the covariance of the projected vector is related to projection of data point onto the eigenvectors of the covariance matrix. To compute the projection vector $\boldsymbol{u}_*$, corresponding to a data point $\boldsymbol{x}_*$, we replace $p(Q \mid X, y)$ with its approximate posterior $q(Q)$ and sample from the following distribution.

$$p\left(\boldsymbol{u}_*, \mid \boldsymbol{x}_*, \boldsymbol{Q}, \boldsymbol{x}, \tilde{\boldsymbol{y}}\right) = \prod_{y=1}^{R} \mathcal{N}\left(\boldsymbol{u}_*, \boldsymbol{\mu}\left(\boldsymbol{q}_s\right)^T \boldsymbol{x}_*, \mathbf{1} + \boldsymbol{x}_*^T \Sigma\left(\boldsymbol{q}_s\right) \boldsymbol{x}_*\right),$$

where $\mu(\cdot)$ and $\Sigma(\cdot)$ denotes the mean vector and covariance matrix for their arguments. In particular, the mean of the projected vector is

$$\boldsymbol{\mu}\left(\boldsymbol{u}_*\right) = \left(\boldsymbol{\mu}\left(\boldsymbol{q}_1\right)^T \boldsymbol{x}_*, \boldsymbol{\mu}\left(\boldsymbol{q}_2\right)^T \boldsymbol{x}_*, \cdots, \boldsymbol{\mu}\left(\boldsymbol{q}_R\right)^T \boldsymbol{x}_*\right).$$

The covariance matrix is given by

$$\begin{aligned} \mathbb{E}\left[\left(\boldsymbol{u}_* - \mu\left(\boldsymbol{u}_*\right)\right)\left(\boldsymbol{u}_* - \mu(\boldsymbol{u})_*\right)^T\right] &= \operatorname{diag}\left(\left(1 + \boldsymbol{x}_*^T \boldsymbol{\Sigma}\left(\boldsymbol{q}_i\right) \boldsymbol{x}_*\right)_{1 \le i \le R}\right) \\ &= \operatorname{diag}\left(\left(1 + \boldsymbol{x}_*^T \left(\operatorname{diag}\left(\boldsymbol{\phi}_i\right) + \boldsymbol{X}\boldsymbol{X}^T\right)^{-1} \boldsymbol{x}_*\right)_{1 \le i \le R}\right). \end{aligned} \tag{2}$$

Now, we write the eigenvalue decomposition of the covariance matrix $\boldsymbol{X}\boldsymbol{X}^T$ corresponding to the data

$$\boldsymbol{X}\boldsymbol{X}^T = \sum_{k=1}^{r} \lambda_k \boldsymbol{v}\boldsymbol{v}^T,$$

where $1 \leq r \leq m$ is the rank. We now have

$$\left(\text{diag}\left(\phi_l\right) + \boldsymbol{X}\boldsymbol{X}^T\right)^{-1} = \sum_{k=1}^{m} \left(\lambda_k + \phi_{ki}\right)^{-1} \boldsymbol{v}\boldsymbol{v}^T. \quad [3]$$

Plugging Eq. 3 into Eq. 2, the covariance matrix can now be rewritten as

$$\begin{aligned}\mathbb{E}\left[\left(u_* - \mu\left(\boldsymbol{u}_*\right)\right)\left(u_* - \mu(\boldsymbol{u})_*\right)\right] &= \text{diag}\left(\left(1 + \boldsymbol{x}_*^T \Sigma\left(\boldsymbol{q}_i\right) \boldsymbol{x}_*\right)_{1 \leq i \leq R}\right) \\ &= \text{diag}\left(\left(1 + \sum_{\mathrm{k}=1}^{m} \left(\lambda_{\mathrm{k}} + \phi_{\mathrm{k}i}\right)^{-1} \left(\boldsymbol{v}^T \boldsymbol{x}_*\right)^2\right)_{1 \leq i \leq R}\right) \\ &= I_{R \times R} + \text{diag}\left(\left(\sum_{k=1}^{m} \left(\lambda_k + \phi_{ki}\right)^{-1} \left(\boldsymbol{v}^T x_*\right)^2\right)_{1 \leq i \leq R}\right).\end{aligned}$$

where $\boldsymbol{I}_{R \times \Omega}$ is the identity matrix. As a result, we observe that, similar to PCA, the covariance of the projected vector $\boldsymbol{u}_*$, is related to the projection of data point $\boldsymbol{x}_*$, onto the orthogonal axes of the covariance matrix $\boldsymbol{X}\boldsymbol{X}^T, i_e, \boldsymbol{v}^T\boldsymbol{x}_*$. In this sense, the projected vector $\boldsymbol{u}_*$, preserves the structure of the data $\boldsymbol{X}\boldsymbol{X}^T$. As the input data $\boldsymbol{X}$ to MDA is set to be mean-subtracted, it also proves that MDA preserves the Global Euclidean distance among the data points.

Preserving Local Structure. In the last step of MDA, a deep neural network trained with uniform manifold approximation and projection (UMAP) (2, 3) loss function is used to embed the projected matrix $\mathbf{U} = \left(\mathbf{u}_1, \cdots, \mathbf{u}_n\right)^T \in \mathbb{R}^{n \times R}$ into $\mathbf{V} = \left(\mathbf{v}_1, \cdots, \mathbf{v}_n\right)^T \in \mathbb{R}^{n \times L}$. A cross entropy loss function defined between distribution of data in the target and embedded spaces (4) is optimized during the training. In particular, the technique computes local, one-directional probabilities $\left(p_{i|j}\right)_{1 \leq i,j \leq n}$ between a point and its k-nearest neighbors to determine the probability with which an edge (or simplex) exists. This is based on the assumption that data are uniformly distributed across a manifold in a warped data space. Under this assumption, a local notion of distance is set by the distance to the k th nearest neighbor, and the local probability is scaled by that local notion of distance, which is defined as:

$$p_{j|i} = \exp\left(-\left(\mathrm{d}\left(\mathbf{u}_i, \mathbf{u}_j\right) - \rho_i\right)/\sigma_i\right). \quad [4]$$

Here, $\mathrm{d}\left(\mathbf{u}_i, \mathbf{u}_j\right)$ represents the distance between the row vectors $\mathbf{u}_i$ and $\mathbf{u}_j$ (e.g., Euclidean distance), σ_i is the standard deviation for the Gaussian distribution, based on the perplexity parameter, such that one standard deviation of the Gaussian kernel fits a set number of nearest neighbors in $\mathbf{U}$. The local connectivity parameter ρ_i is set to the distance from x_i to its nearest neighbor, and σ_i is set to match the local distance around $\mathbf{u}_i$ upon its k nearest neighbors (where k is a hyperparameter). After computing the one-directional edge probabilities for each data point, a global probability is computed as the probability of either of the two local, one-directional probabilities occurring, which is defined as:

$$p_{ij} = \left(p_{j|i} + p_{i|j}\right) - p_{j|i}p_{i|j}. \quad [5]$$

The computation of the pairwise probability q_{ij} between points in the embedding space $\mathbf{V} = \left(\mathbf{v}_1, \cdots, \mathbf{v}_n\right)^T \in \mathbb{R}^{n \times L}$ uses the following function:

$$q_{ij} = \left(1 + a\left\|\mathbf{v}_i - \mathbf{v}_j\right\|^{2b}\right)^{-1}, \quad [6]$$

where a and b are hyperparameters that are set based on a desired minimum distance between points in the embedding space. To find the embedded vectors $\mathbf{v}_1, \cdots, \mathbf{v}_n$, a cross entropy loss function is optimized. In particular, the following loss function is defined

$$H(P, Q) = \sum_{i \neq j} p_{ij} \log\left(\frac{p_{ij}}{q_{ij}}\right) + \left(1 - p_{ij}\right) \log\left(\frac{1 - p_{ij}}{1 - q_{ij}}\right), \quad [7]$$

where $P = (p_{ij})_{1 \leq i,j \neq n}$, and $Q = (q_{ij})_{1 \leq i,j \leq n}$. Like UMAP and t-SNE, the optimization above (minimization of H) preserves the local data structure or local distance of the data points. See Ref. (2) for details on how minimization of H preserves the local data structure. As such, MDA emerges as an optimal blend of global structure-preserving techniques like PCA and MDS, and local structure-preserving methods like t-SNE, UMAP and LLE.

4. Relationship between PCA and MDS

Both Principal Component Analysis (PCA) and Classical Multidimensional Scaling (MDS) are techniques used for dimensionality reduction and data visualization. PCA identifies the axes along which the variance of the data is maximized. Mathematically, it involves diagonalizing the covariance matrix of the data and projecting the data onto the principal components (eigenvectors). MDS aims to map the original data points into a lower-dimensional space while preserving the pairwise distances among the points as much as possible. It typically starts with a distance matrix $\mathbf{D}$ and works to find a configuration of points in the lower-dimensional space that approximates these distances.

The relationship between PCA and MDS can be understood through the following points:

1. Distance Matrix and Covariance Matrix: If the distance matrix $\mathbf{D}$ in MDS is generated from a set of centered points (i.e., mean of each feature is zero), then the double-centered distance matrix $\mathbf{B}$ in MDS is related to the covariance matrix $\mathbf{\Sigma}$ in PCA. Specifically, $\mathbf{B} = -\frac{1}{2}\mathbf{HDH}$, where $\mathbf{H}$ is the centering matrix, and $\mathbf{B}$ and $\mathbf{\Sigma}$ are related up to a scaling factor and sign.

2. Eigenvectors: The eigenvectors $\mathbf{u}$ of $\mathbf{B}$ in MDS are the same as the eigenvectors $\mathbf{v}$ of $\mathbf{\Sigma}$ in PCA.

3. Eigenvalues: The eigenvalues α of $\mathbf{B}$ in MDS are related to the eigenvalues λ of $\mathbf{\Sigma}$ in PCA by $\alpha = n\lambda$, where n is the number of data points.

Thus, PCA and classical MDS can be equivalent when the distance matrix used in MDS is a Euclidean distance matrix and the data points from which the distance matrix is derived are centered. In this case, both methods will yield the same coordinates for the points in the reduced-dimensional space.

Detailed proof of relationship between PCA and MDS. Let's establish the relationship between the eigenvalues and eigenvectors of $\mathbf{B}$ in MDS and $\mathbf{\Sigma}$ in PCA mathematically.

Let $\mathbf{X}$ be a $n \times d$ data matrix where n is the number of data points and d is the number of dimensions. Assume that $\mathbf{X}$ is centered, meaning that the mean of each column is zero. $\mathbf{D}$ is the $n \times n$ squared distance matrix, where $\mathbf{D}_{ij} = \|x_i - x_j\|^2$. $\mathbf{B}$ is the double-centered distance matrix in MDS, calculated as $\mathbf{B} = -\frac{1}{2}\mathbf{HDH}$, where $\mathbf{H} = \mathbf{I} - \frac{1}{n}\mathbf{1}\mathbf{1}^T$ and $\mathbf{1}$ is an $n \times 1$ vector of ones. $\mathbf{\Sigma}$ is the $d \times d$ covariance matrix in PCA, calculated as $\mathbf{\Sigma} = \frac{1}{n}\mathbf{X}^T\mathbf{X}$.

1. Expression for B:

We start by expressing $\mathbf{B}$ in terms of $\mathbf{X}$:

$\mathbf{D}ij = \|x_i - x_j\|^2 = (x_i - x_j)^T(x_i - x_j) = x_i^T x_i - 2x_i^T x_j + x_j^T x_j$

$\mathbf{B} = -\frac{1}{2}\mathbf{HDH}$

Expanding $\mathbf{B}$ in terms of $\mathbf{X}$ using the above formula for $\mathbf{D}$, and applying the centering matrix $\mathbf{H}$, we get:

$\mathbf{B} = \mathbf{XX}^T$

2. Eigen-decomposition of B and Σ:

Let's say $\mathbf{B}$ and $\mathbf{\Sigma}$ have the following eigen-decompositions:

$\mathbf{Bu} = \alpha\mathbf{u}$ and $\mathbf{\Sigma v} = \lambda\mathbf{v}$

Since $\mathbf{B} = \mathbf{XX}^T$ and $\mathbf{\Sigma} = \frac{1}{n}\mathbf{X}^T\mathbf{X}$, we can rewrite these equations as:

$\mathbf{XX}^T\mathbf{u} = \alpha\mathbf{u}$ and $\frac{1}{n}\mathbf{X}^T\mathbf{Xv} = \lambda\mathbf{v}$

3. Relationship between α and λ:

Multiply both sides of the second equation by $\mathbf{X}$:

$$\mathbf{X}\left(\frac{1}{n}\mathbf{X}^T\mathbf{X}\right)\mathbf{v} = \mathbf{X}(\lambda\mathbf{v}) \quad [8]$$

$$\Rightarrow \frac{1}{n}\mathbf{XX}^T(\mathbf{Xv}) = \lambda(\mathbf{Xv}) \quad [9]$$

Comparing this with the eigen-equation for $\mathbf{B}$, $\mathbf{XX}^T\mathbf{u} = \alpha\mathbf{u}$, we find that $\mathbf{u} = \mathbf{Xv}$) and $\alpha = n\lambda$.

Thus, under the conditions of centered data and Euclidean distances, we find that:

- The eigenvectors $\mathbf{u}$ of $\mathbf{B}$ are related to the eigenvectors $\mathbf{v}$ of
- The eigenvalues α of $\mathbf{B}$ are related to the eigenvalues λ of $\mathbf{\Sigma}$ by $\alpha = n\lambda$.

5. Distribution of distance values in different tasks

A folded Gaussian distribution (see Fig. S3) is only a good *approximation* for the true underlying distribution that is defined by the geodesic distance between data points. The true underlying distribution indeed depends on the sampling procedure of the training dataset as well the underlying manifold geometry. For instance, on a compact data manifold, the distance between data points is distributed on a closed interval $[0, D]$, where

$$D = \min_{p,q} d(p, q).$$

As a result, a folded Gaussian distribution whose support is $[0, \infty)$ can only be an approximation for the distribution of geodesic distance of data points that are defined by a compact geometry. Nevertheless, as it is evident from Figs. S4-S7, for

practical datasets, such a folded Gaussian distribution can provide a good estimate for the width of bins in the histogram bin count method. Moreover, similar to the original Scott's optimal histogram binning method (5), in our case, the choice of folded Gaussian distribution leads to the following bin width (see Equation 12 in the paper)

$$h_m^* = 3^{1/3}\pi^{1/6}\sigma((m^2 - m)/2)^{-1/3}. \quad [10]$$

which can be shown to be a good estimate for many distributions with a positive support. To show that h_m^* is indeed a good estimate for a large class of densities, we follow a similar argument as in Ref. (5), whereby we consider the optimal bin width for folded Gaussian density and non-Gaussian densities with equal variances and positive support, and observe how their theoretically optimal bin widths differ. In particular, we considered three models of non-Gaussian behavior: skewed, heavy-tailed and bimodal densities. In Fig. S8 we show the ratio between the bin width estimate $\underline{h}_m^*$ and the optimal value in Eq. 10. The ratio can be expressed as

$$\text{Ratio}\ (\theta) = \frac{3^{1/3} \times \pi^{1/6} \times \sigma \times \left(\left(m^2 - m\right)/2\right)^{-1/3}}{\left(\frac{6}{\int_\sigma^\infty (p(s;\theta))^2 ds}\right)^{1/3} \times \left(\left(m^2 - m\right)/2\right)^{-1/3}},$$

Where θ is the parameter of the distribution (e.g., the skewness coefficient). From Fig. S8, we see that this ratio is near 1 for a wide range of parameter θ in each case, indicating that the estimated bin widths h_m^* is a sufficiently good approximation for many densities.

Quantitative Assessment of 'Folded Gaussian' Fit Quality. To bolster the argument that a 'folded Gaussian' is a suitable model for representing distances across the manifold, we applied curve fitting to distance histograms for DNN features extracted from three distinct datasets (codes are added in Code Ocean capsule for one dataset). We utilized various probability distribution models for the curve fitting. The results clearly indicate that the 'folded Gaussian' model yields the most accurate fit, as evidenced by the lowest Root Mean Square Error (RMSE) as shown in Figs. S4-S7.

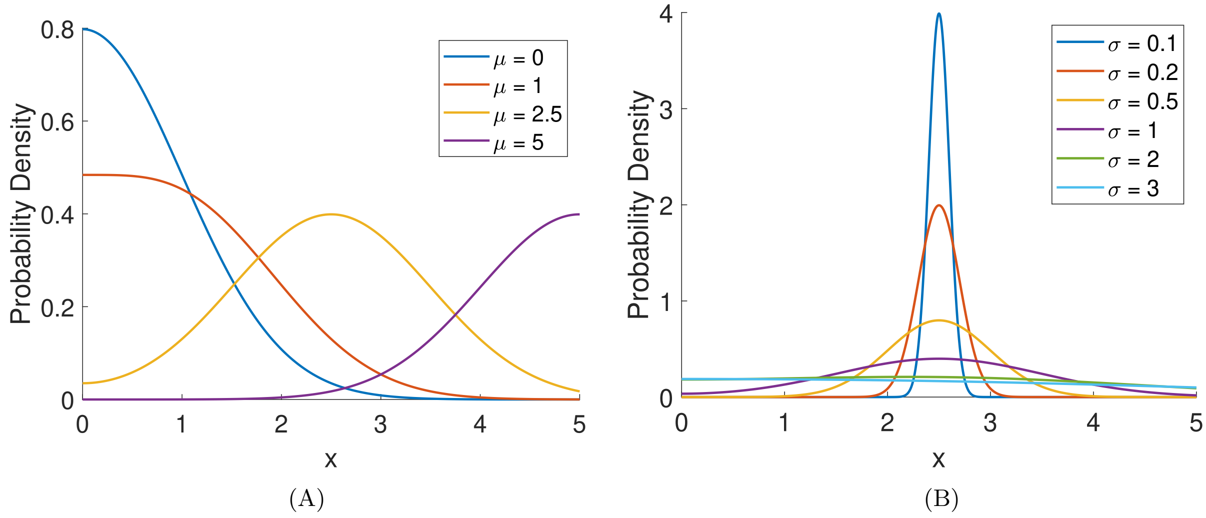

Fig. S3. Folded Gaussian distributions for (A) different means and standard deviation (σ) of 1, (B) mean of 2.5 and different standard deviations. Source data are provided as a Source Data file.

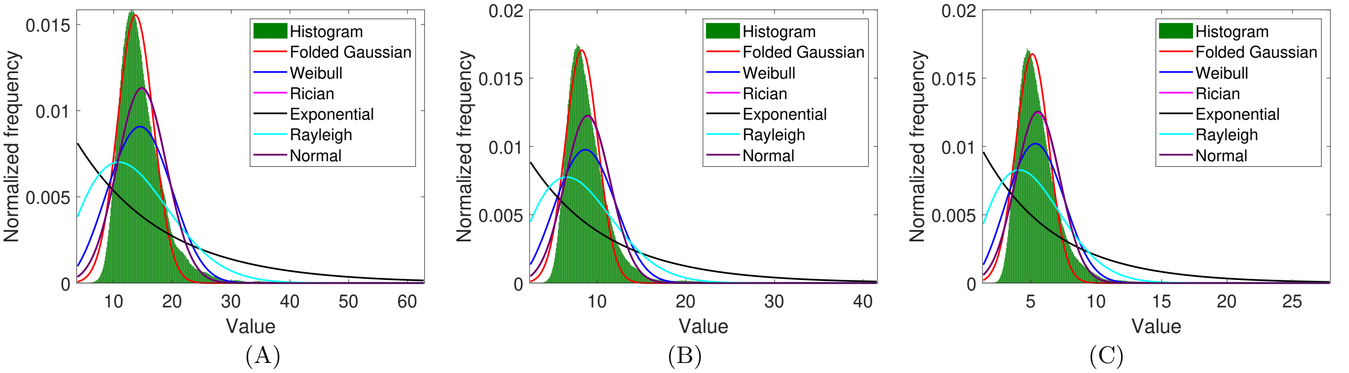

Fig. S4. Fitted curves to histograms of distances among the extracted features of layer number 6 (A), 8 (B) and 10 (C) of fMLP (Table S6) from TCGA dataset. Source data are provided as a Source Data file.

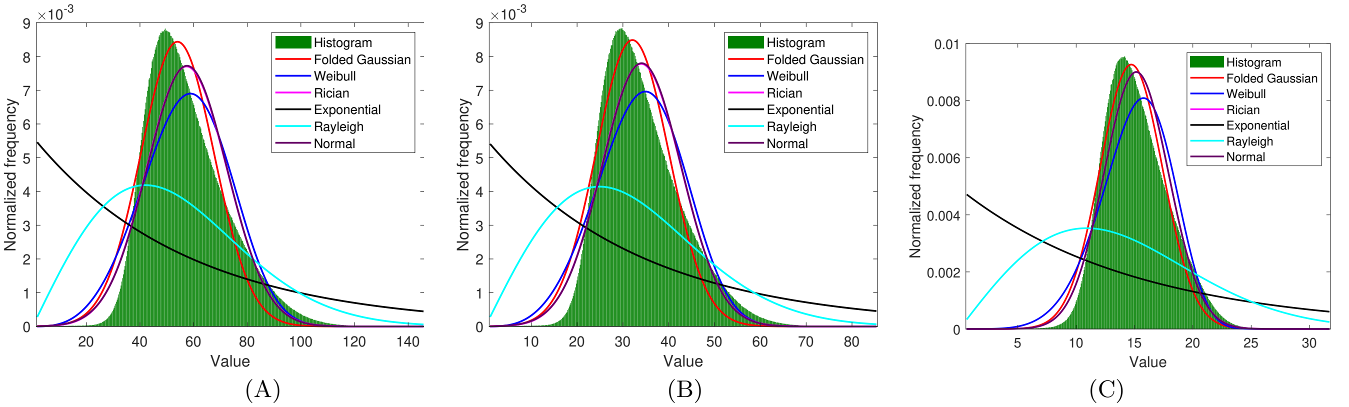

Fig. S5. Fitted curves to histograms of distances among the extracted features of layer number 10 (A), 12 (B) and 14 (C) of mCNN (Table S5) from MNIST dataset. Source data are provided as a Source Data file.

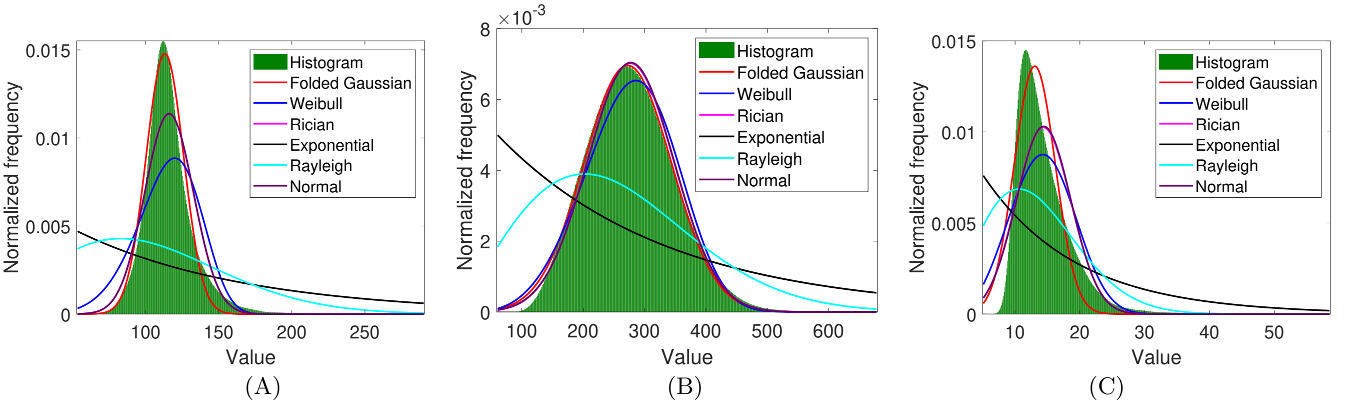

Fig. S6. Fitted curves to histograms of distances among the extracted features of layer number 160 (A), 174 (B) and 181 (C) of DarkNet (6) from diabetic retinopathy (DR) dataset. Source data are provided as a Source Data file.

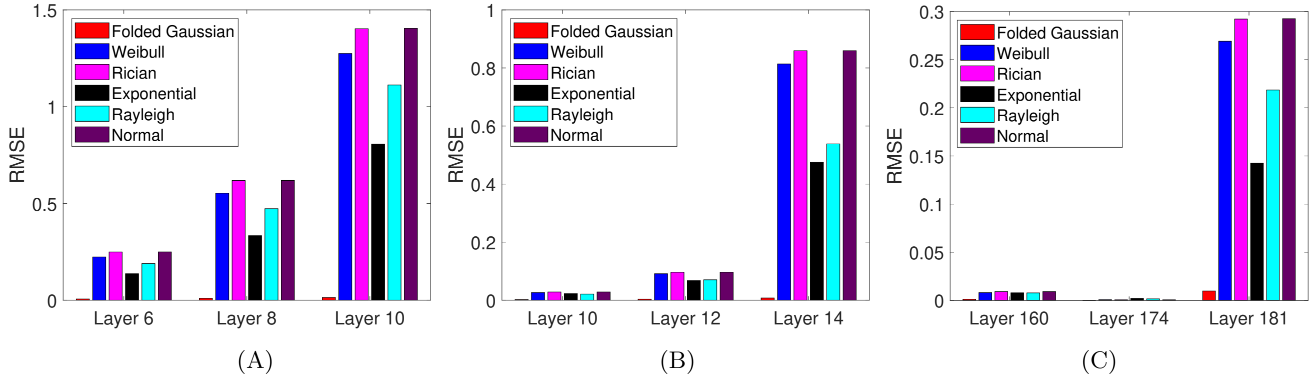

Fig. S7. RMSEs of the curve fitting for (A) TCGA, (B) MNIST and (C) DR dataset. Source data are provided as a Source Data file.

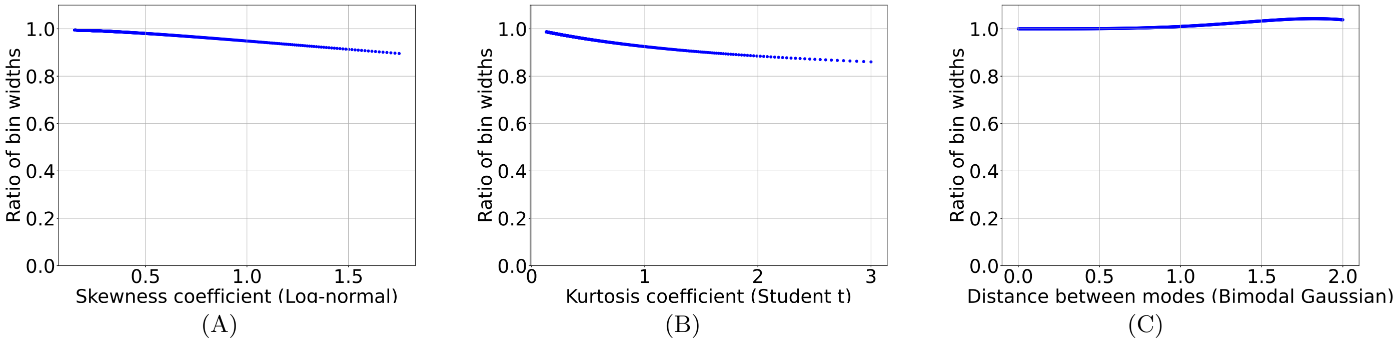

Fig. S8. Ratio of bin width of a folded Gaussian distribution to non-Gaussian densities. (A) Log-normal (B) Student t (C) Bimodal Gaussian. Source data are provided as a Source Data file.

6. Visualization of features for a deep regression task

A simple regression problem with MNIST hand-written digits is studied here to shed useful insights into the MDA visualization of deep regression (Fig. S9). The inputs are hand-written digit images (0 to 9) rotated by varying angles within the range of -45 to +45 degrees (see examples in Fig. S10), and the task is to predict the rotation angle. We trained a deep network with 19 layers, including input neurons, convolutional, batch normalization, ReLU (rectified linear unit), average pooling, dropout, and fully connected layers (a). The PCA, t-SNE, and UMAP visualization of the training data features are shown in (b) at layers 4, 8, 12, and 15. Except for the PCA results at layer 15, which display a continuous variation of data from -45 to +45 degrees to some extent (e1), all other visualizations provide no useful information, with data points mostly distributed randomly. The MDA visualizations at the layers are displayed in (b-last row). It can be observed that in the initial layer (layer 2), there is already a continuity in data points from red (representing +45 degrees) to violet (representing -45 degrees). However, numerous dislocated points in this continuous path gradually improve as we delve deeper into the DNN. In the last two layers (12 and 15), continuous variations of the feature data are clearly visible. The results make sense, as the data labels are continuous and the feature space data of a well-trained DNN should reflect this relationship. MDA's superior results are also evident in the model test (Fig. S11 (a1-e4)).

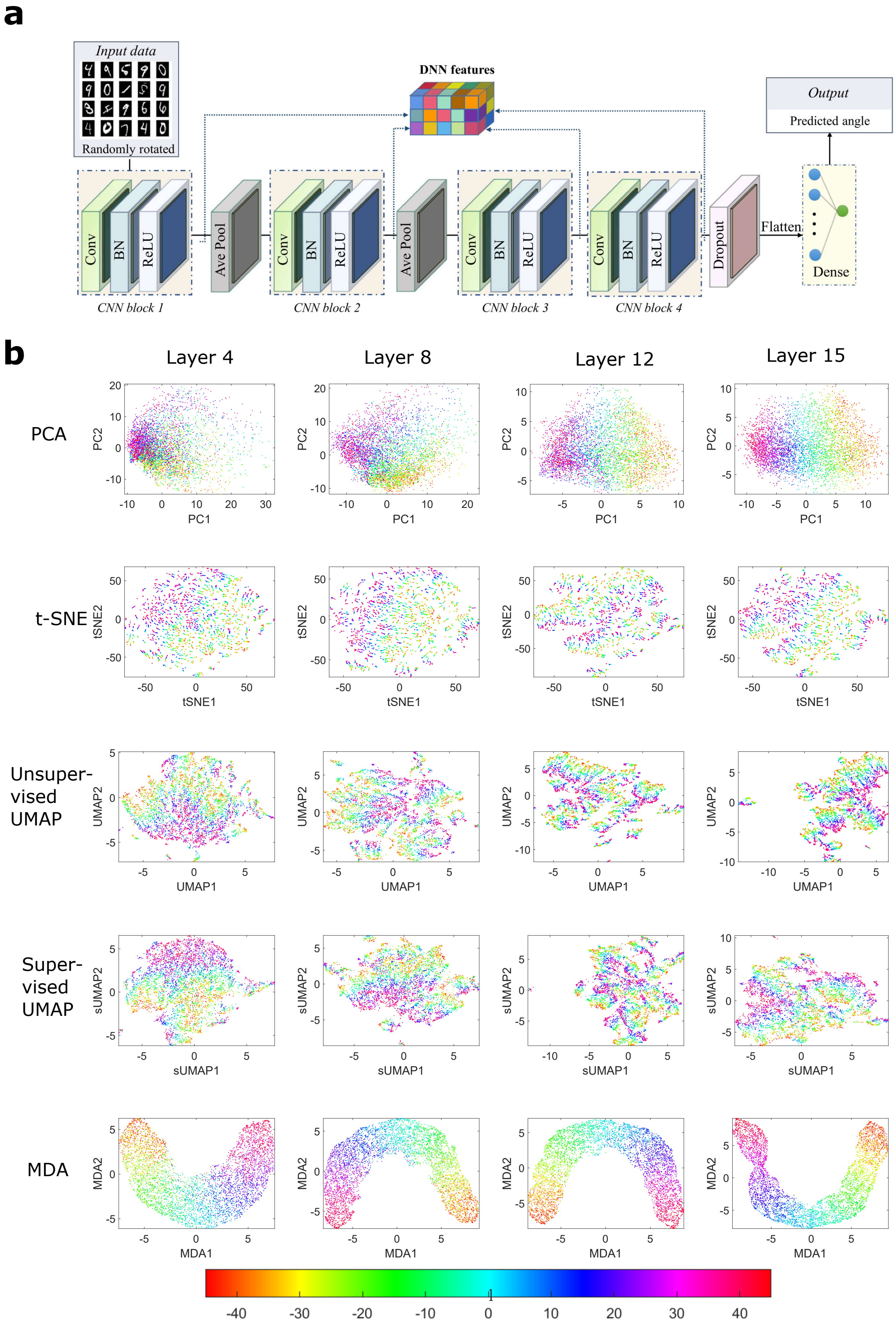

Fig. S9. Estimation of the angle of digits from MNIST Dataset (7). (a) The employed DNN architecture. (b) PCA, t-SNE, unsupervised and supervised UMAP, and MDA visualizations of the DNN features of training data at layers 4, 8, 12, and 15. Different colors represent different angles, starting with red (corresponding to data at -45 degrees) and ending with violet (corresponding to data at +45 degrees). Source data are provided as a Source Data file.

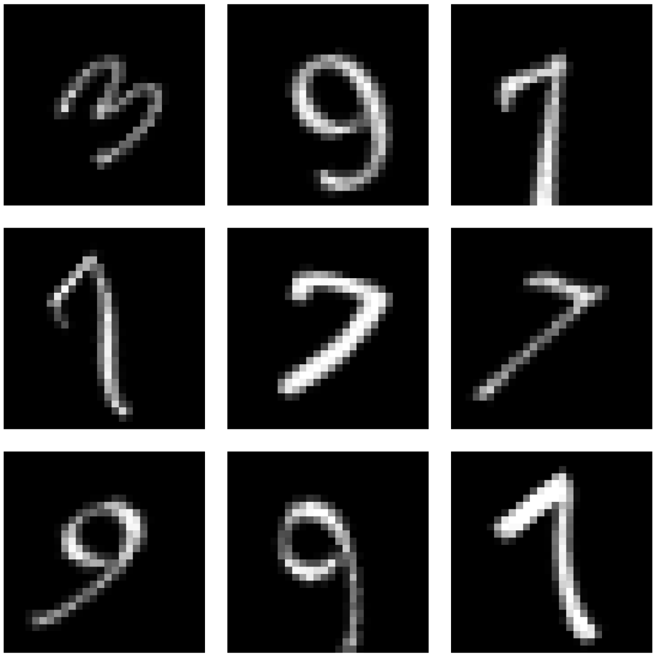

(a)

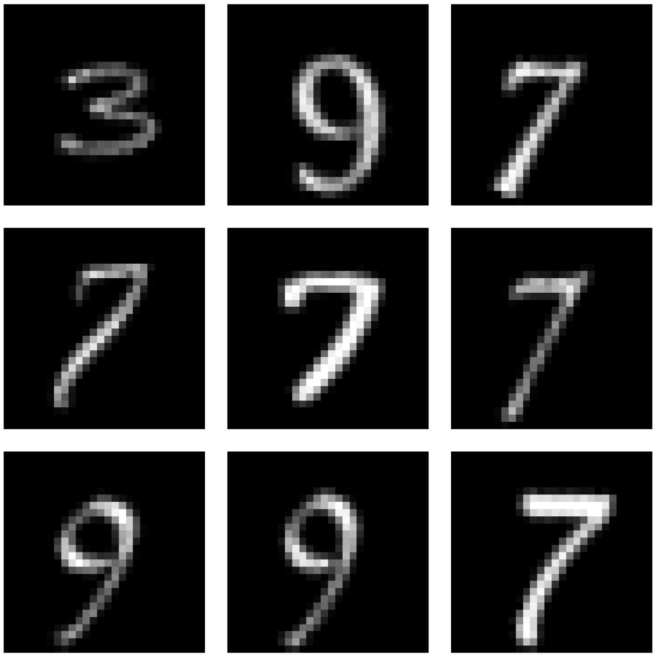

(b)

Fig. S10. Example of digit images used in analyses of Fig. S9. (a) Rotated digit images at different angles. (b) Corresponding unrotated digit images.

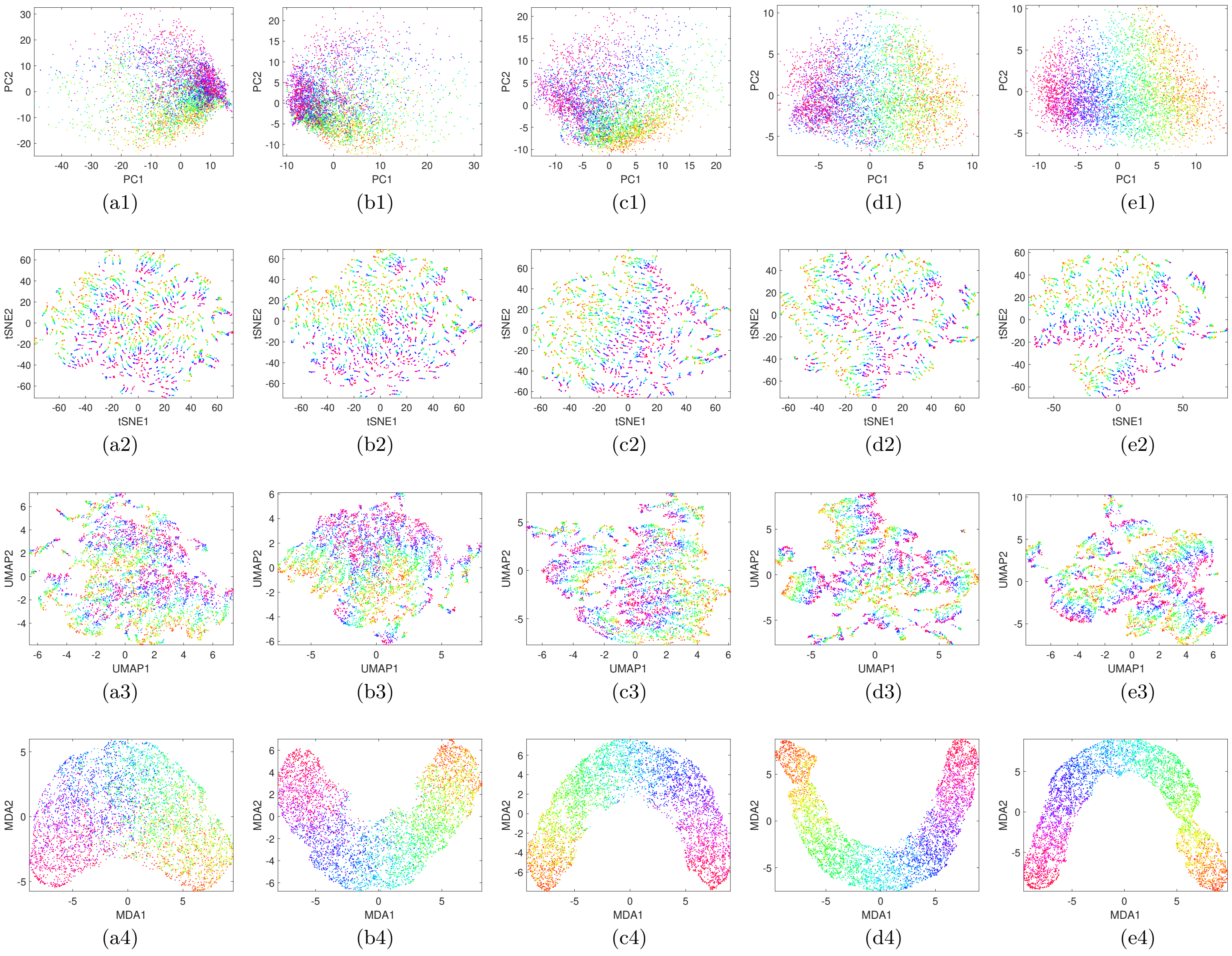

Fig. S11. Continuous prediction problem regarding finding the angle of digits from -45 to $+45$ degree. PCA (1), t-SNE (2), UMAP(3) and MDA (4) visualizations of the DNN features of test data at layers 2(a), 4(b), 8(c), 12(d) and 16(e). Different colors represent different angles starting from red, which corresponds to the data of -45 degree and ending at violet, which corresponds to data of $+45$ degree. Source data are provided as a Source Data file.

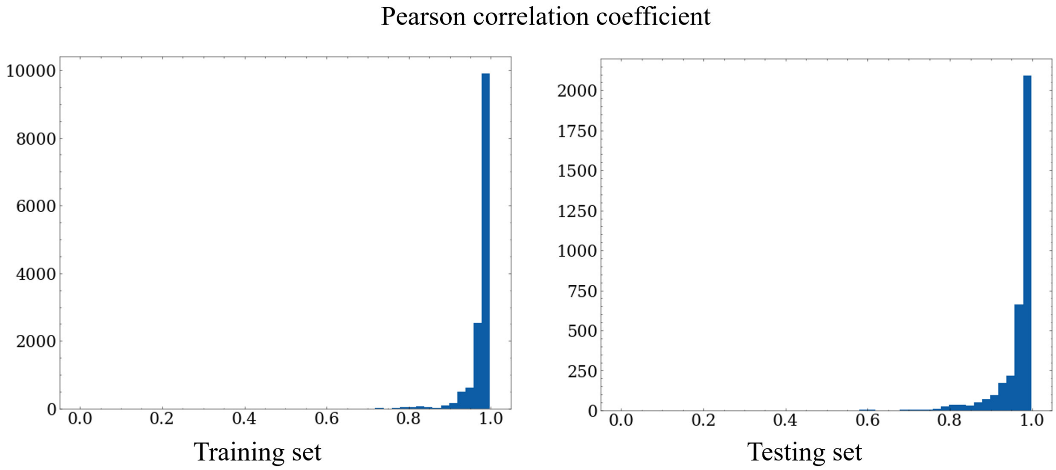

Fig. S12. Pearson correlation coefficient plots of training and testing set in gene expression prediction task. Source data are provided as a Source Data file.

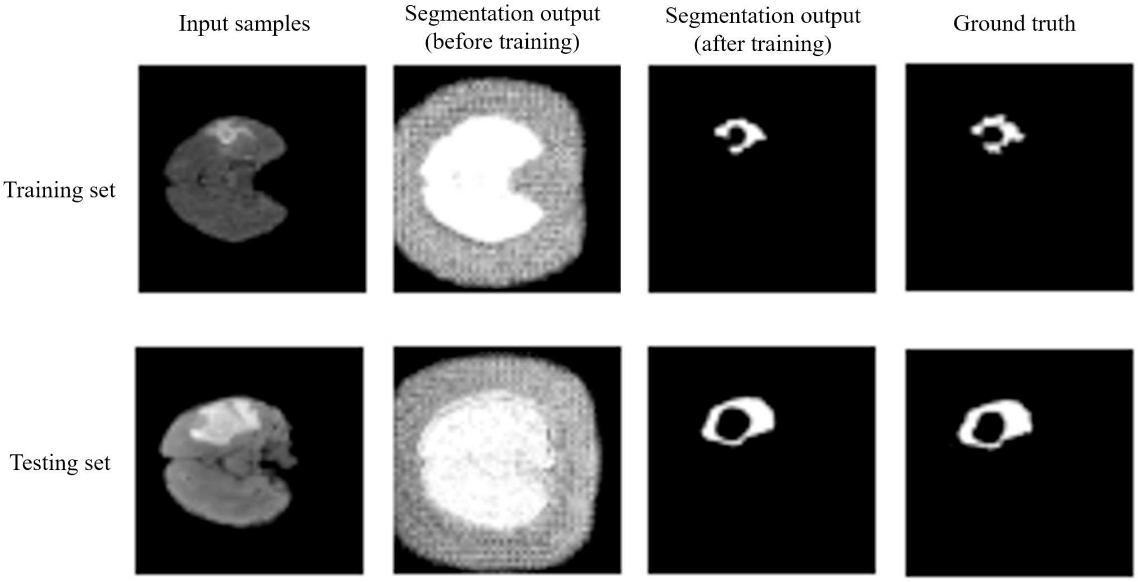

Fig. S13. Segmentation results of the training and testing sets.

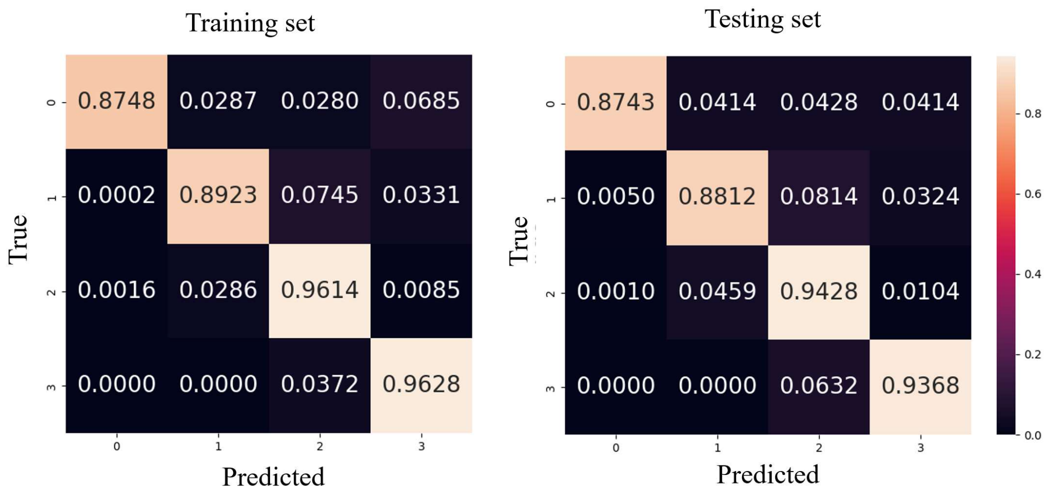

Fig. S14. Confusion matrices of the classification results.

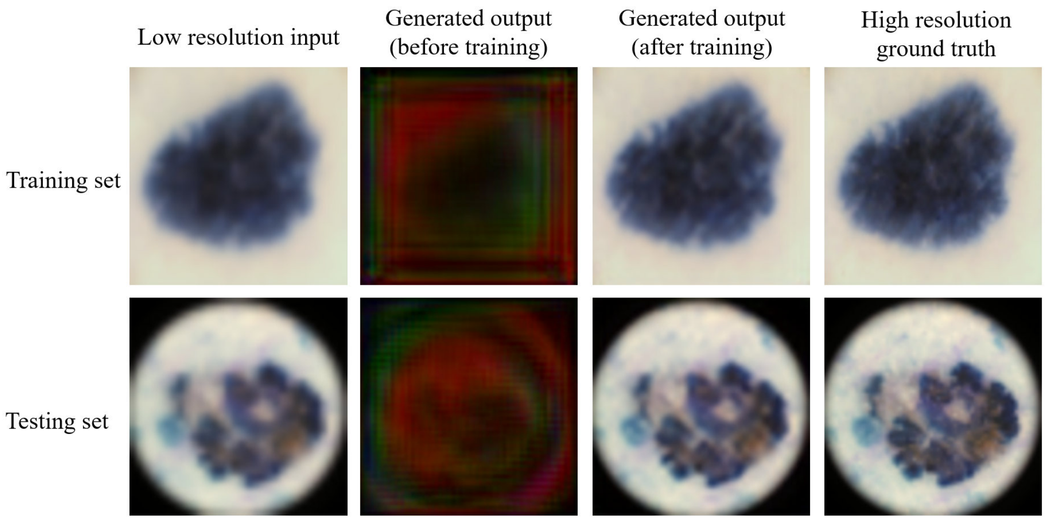

Fig. S15. Examples of the SRGAN results.

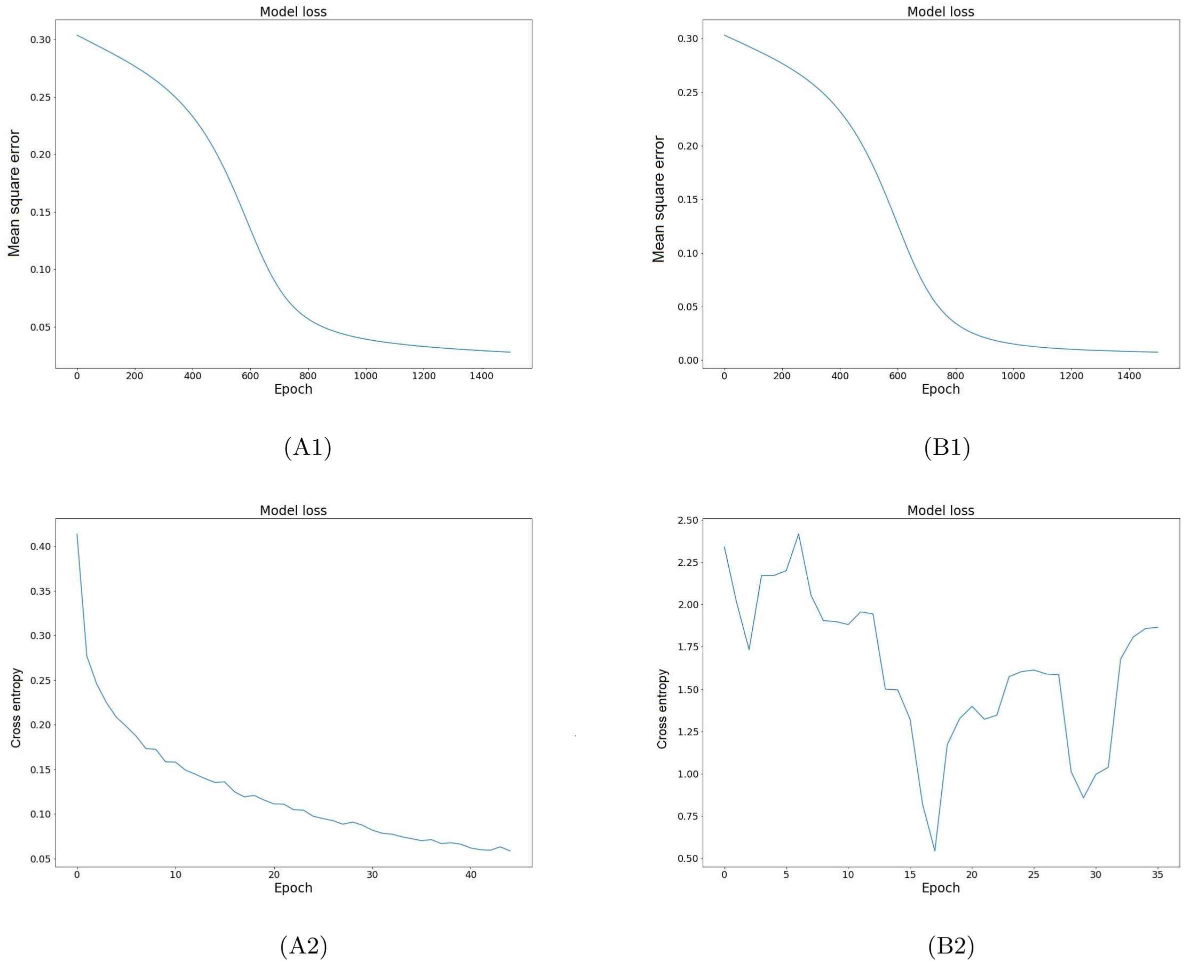

Fig. S16. Training loss (A) and validation loss (B) for gene expression prediction (1) and classification tasks (2). Source data are provided as a Source Data file.

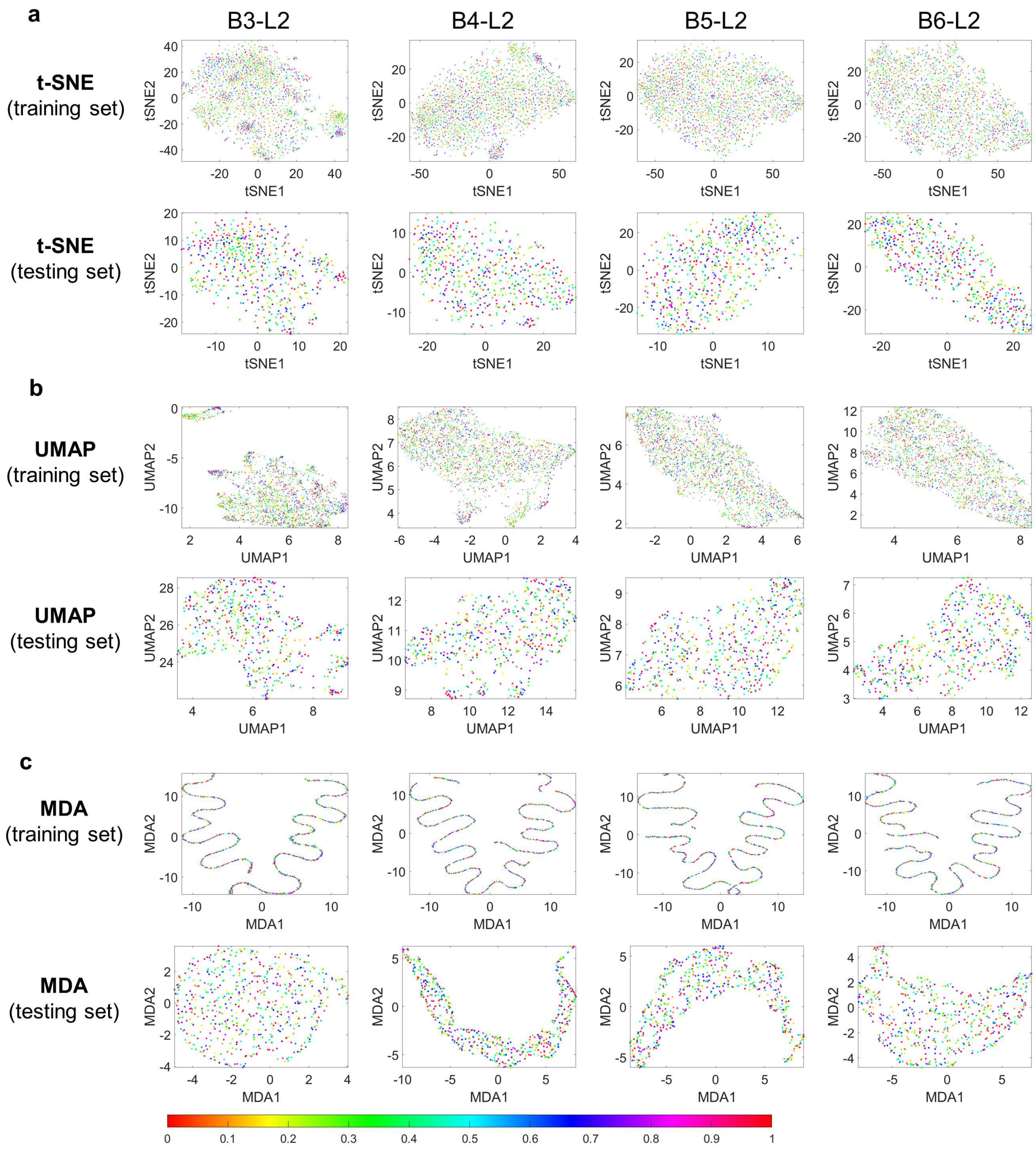

Fig. S17. Visualization of DNN features before network training by (a) t-SNE, (b) UMAP, and (c) MDA for survival prediction. Source data are provided as a Source Data file.

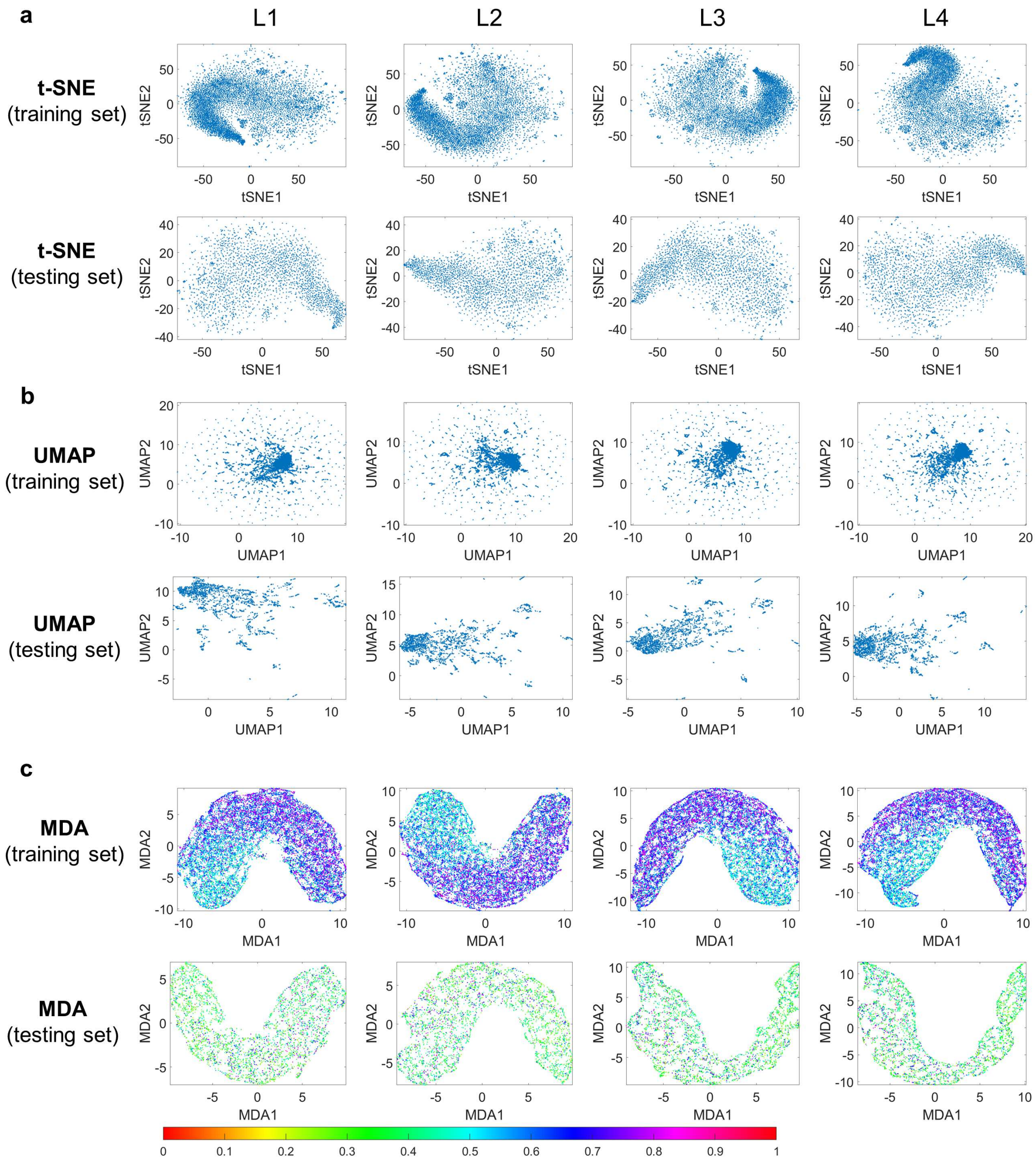

Fig. S18. Visualization of DNN features before network training by (a) t-SNE, (b) UMAP, and (c) MDA for gene expression prediction. Source data are provided as a Source Data file.

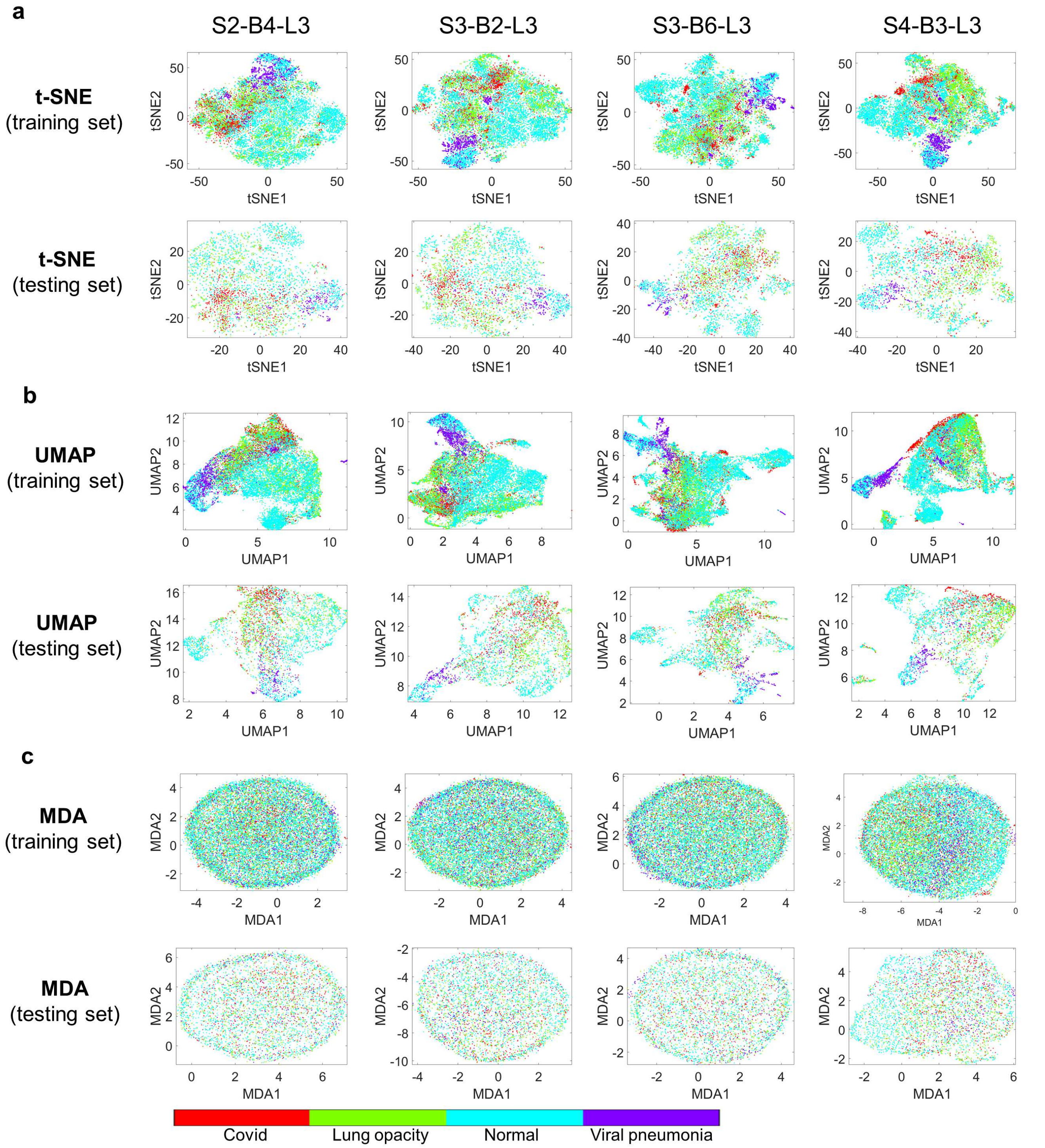

Fig. S19. Visualization of DNN features before network training by (a) t-SNE, (b) UMAP, and (c) MDA for COVID-19 data classification. Source data are provided as a Source Data file.

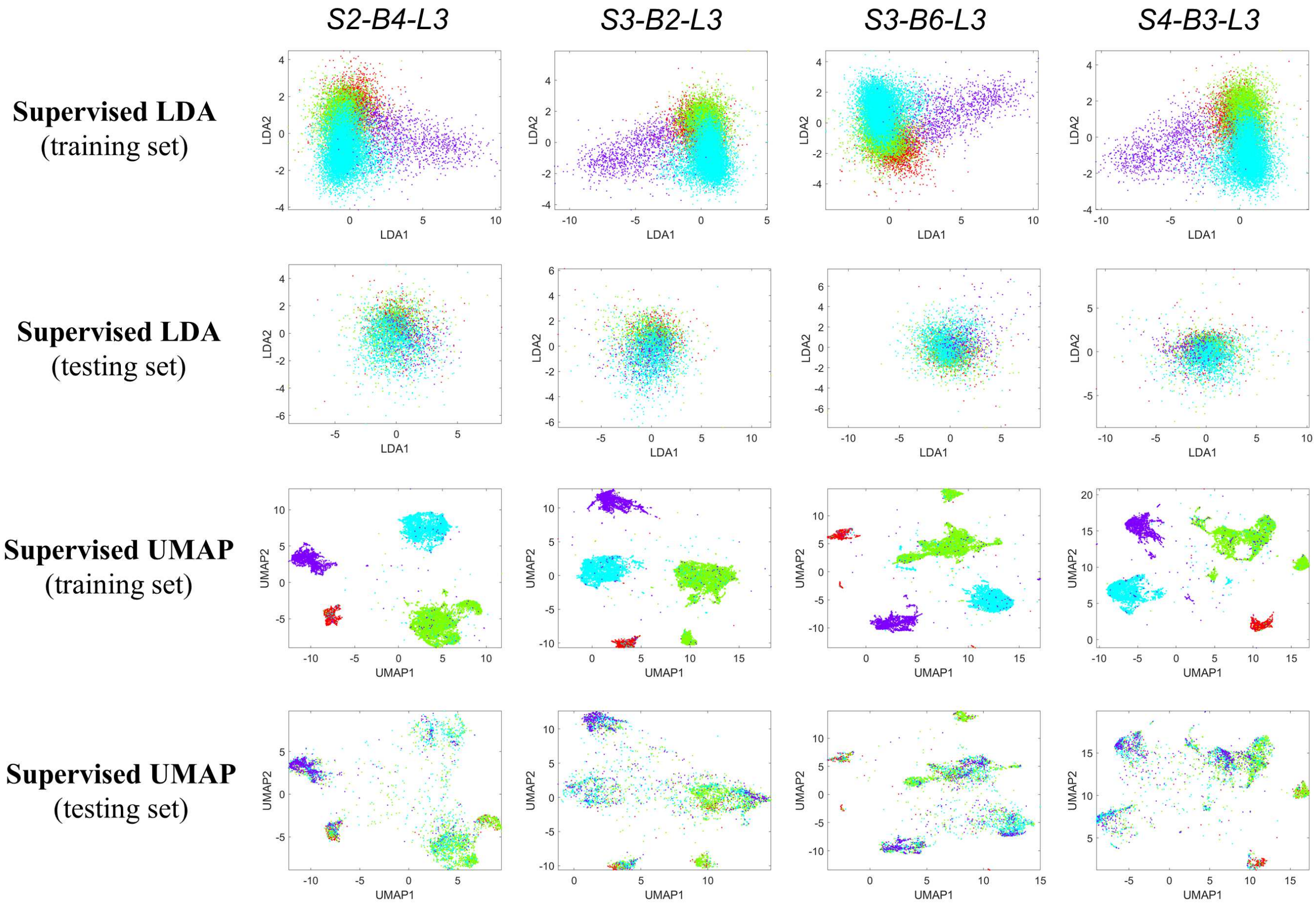

Fig. S20. Visualization of DNN features before network training by Supervised LDA and UMAP for COVID-19 data classification. See Fig. S19 for the colormap. Source data are provided as a Source Data file.

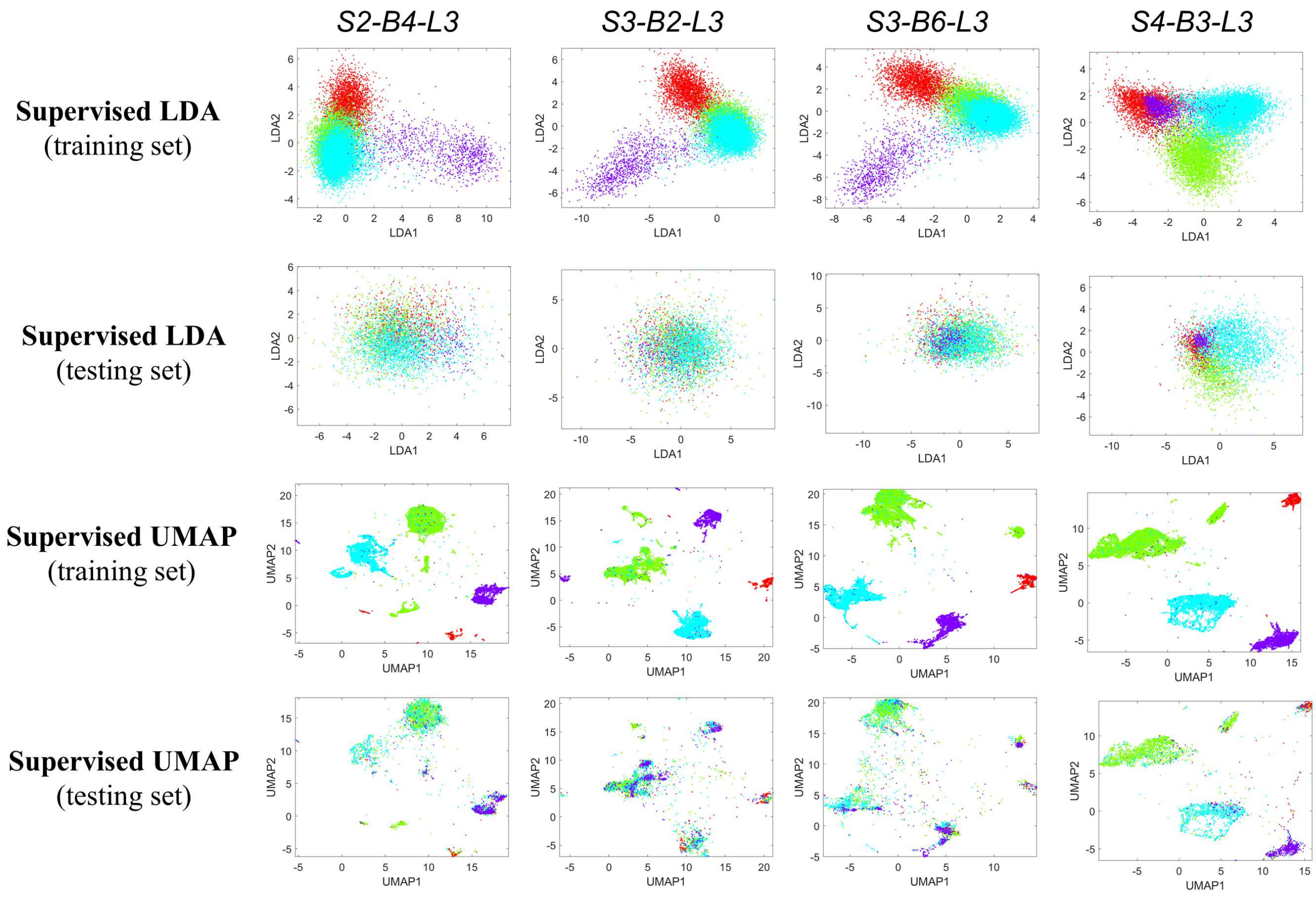

Fig. S21. Visualization of DNN features after network training by Supervised LDA and UMAP for COVID-19 data classification. See Fig. S19 for the colormap. Source data are provided as a Source Data file.

7. Visualization of features at intermediate epochs

In this experiment, we applied MDA to investigate the feature visualization differences at different epochs of the training for two DNNs. We selected the intermediate testing data features of the Dense-UNet and ResNet for segmentation and classification tasks at different training epochs and visualized them in Fig. S22 (a). We chose the features of the training and testing sets at epoch=1, 3 and 10. Our results showed that the visualization of the features at epoch=1 and 3 appeared more disordered in color compared to that of epoch=10. This observation indicates that our visualization method is capable of reflecting the network's training status. For classification tasks, the data points corresponding to different classes gradually become separated with epochs (Fig. S22 b).

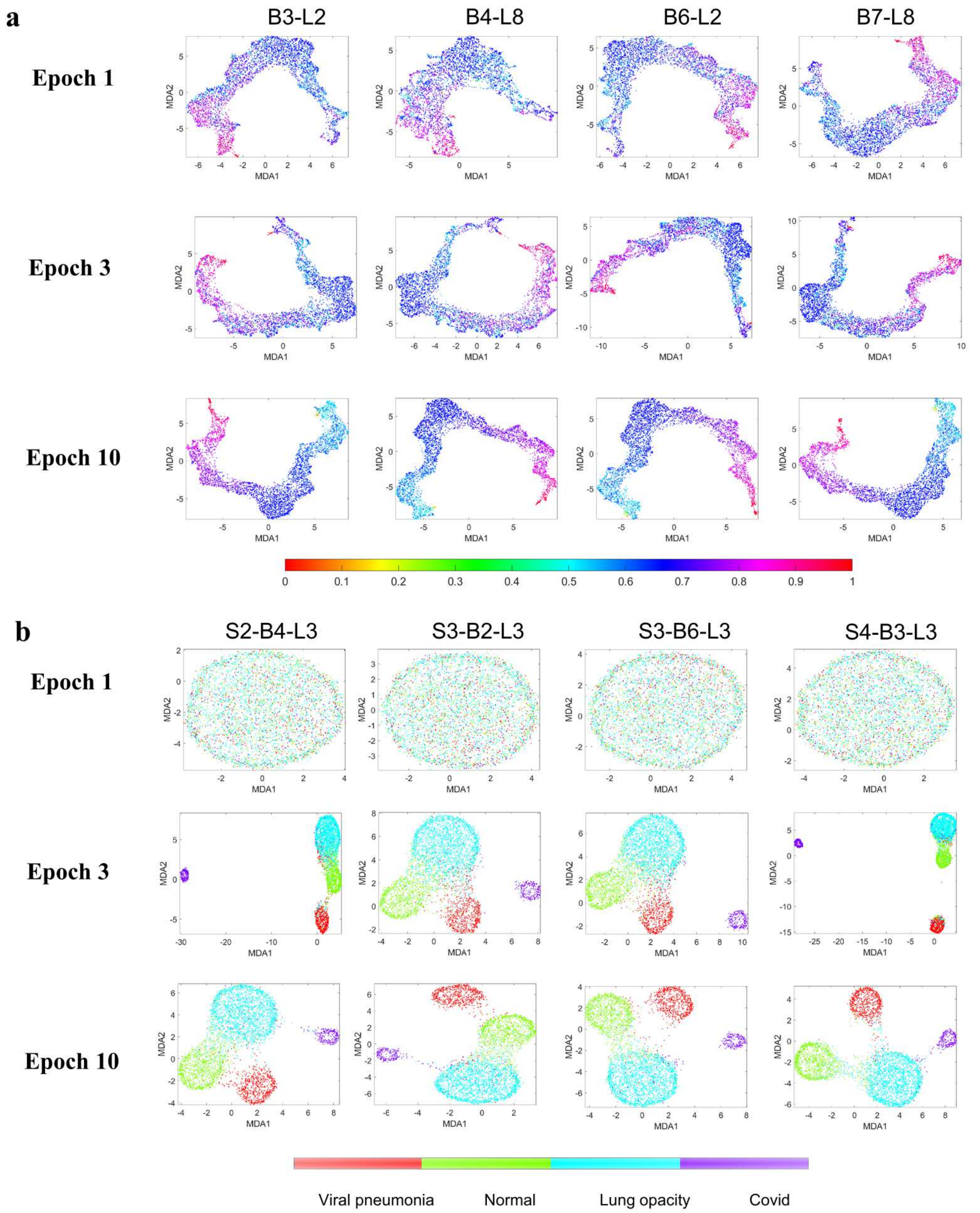

Fig. S22. MDA visualization of the feature space of (a) Dense-UNet and (b) ResNet trained on BraTS and COVID-19 dataset for segmentation and classification with progression of epochs. Source data are provided as a Source Data file.

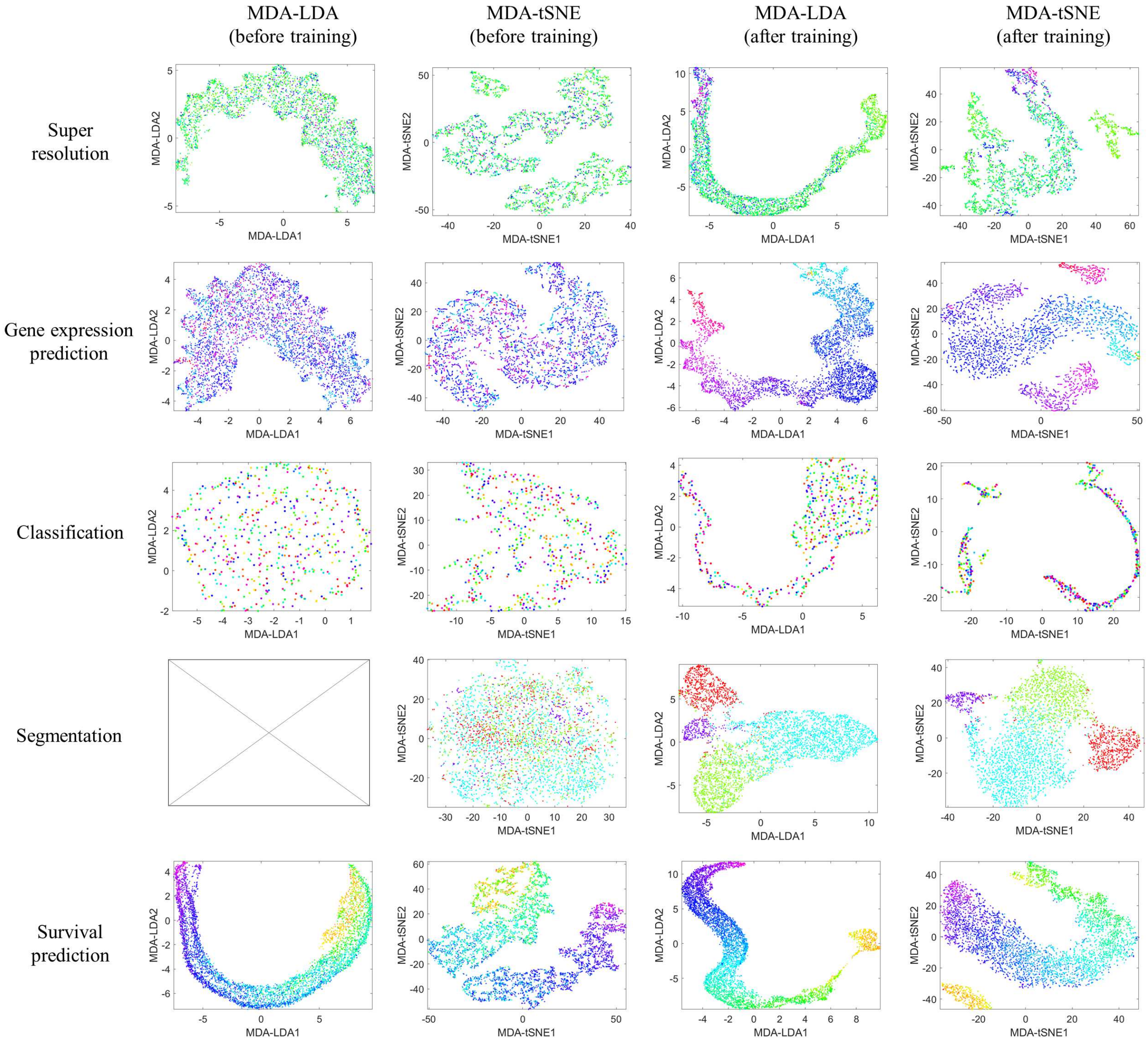

Fig. S23. Ablation study showing the effect of replacing Bayesian projection and deep learning embedding of MDA by LDA and t-SNE, respectively in visualizations of features from different DNNs for different tasks. The layers used here for visualizations are the last layers of the DNNs used in different tasks (see the main manuscript for the colormaps). Source data are provided as a Source Data file.

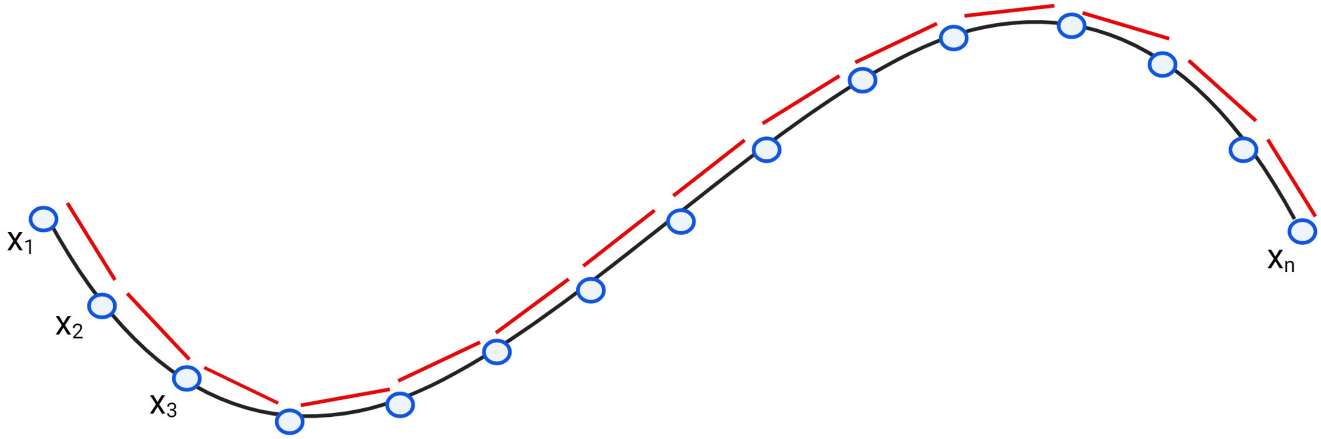

Fig. S24. Demonstration of how preservation of the Euclidean distance in small parts of the manifold in MDA preserves the geodesic distance among the data points. The red line denotes the Euclidean distance between two nearby points.

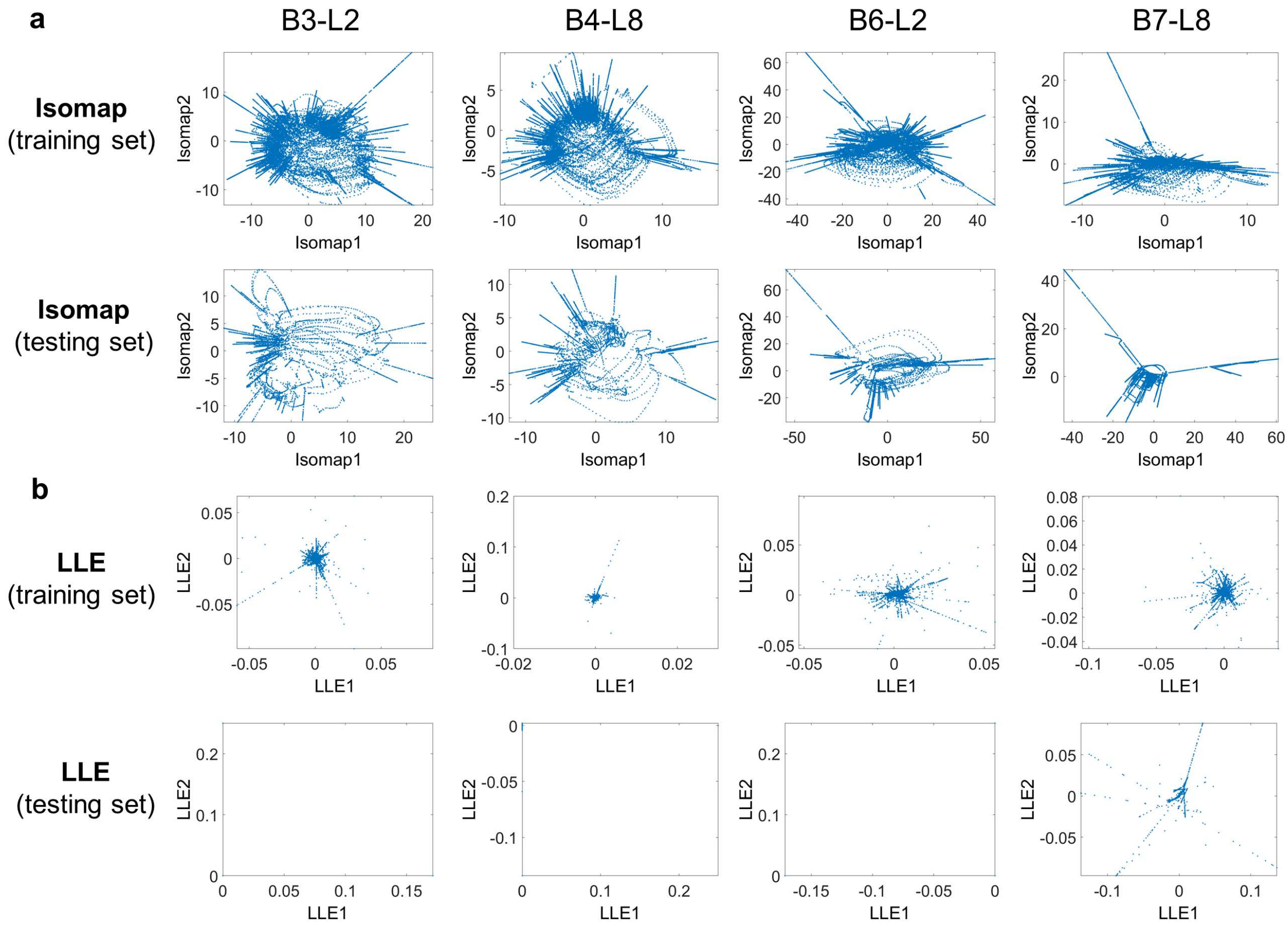

Fig. S25. Visualizations of the Dense-UNet features for segmentation task before training the network by (a) Isomap and (b) LLE. Here, B3-L2 denotes the 2nd layer of the 3rd dense block, B4-L8 denotes the 8th layer of the 4th dense block, B6-L2 denotes the 2nd layer of the 6th dense block, and B7-L8 denotes the last layer before the final output. Source data are provided as a Source Data file.

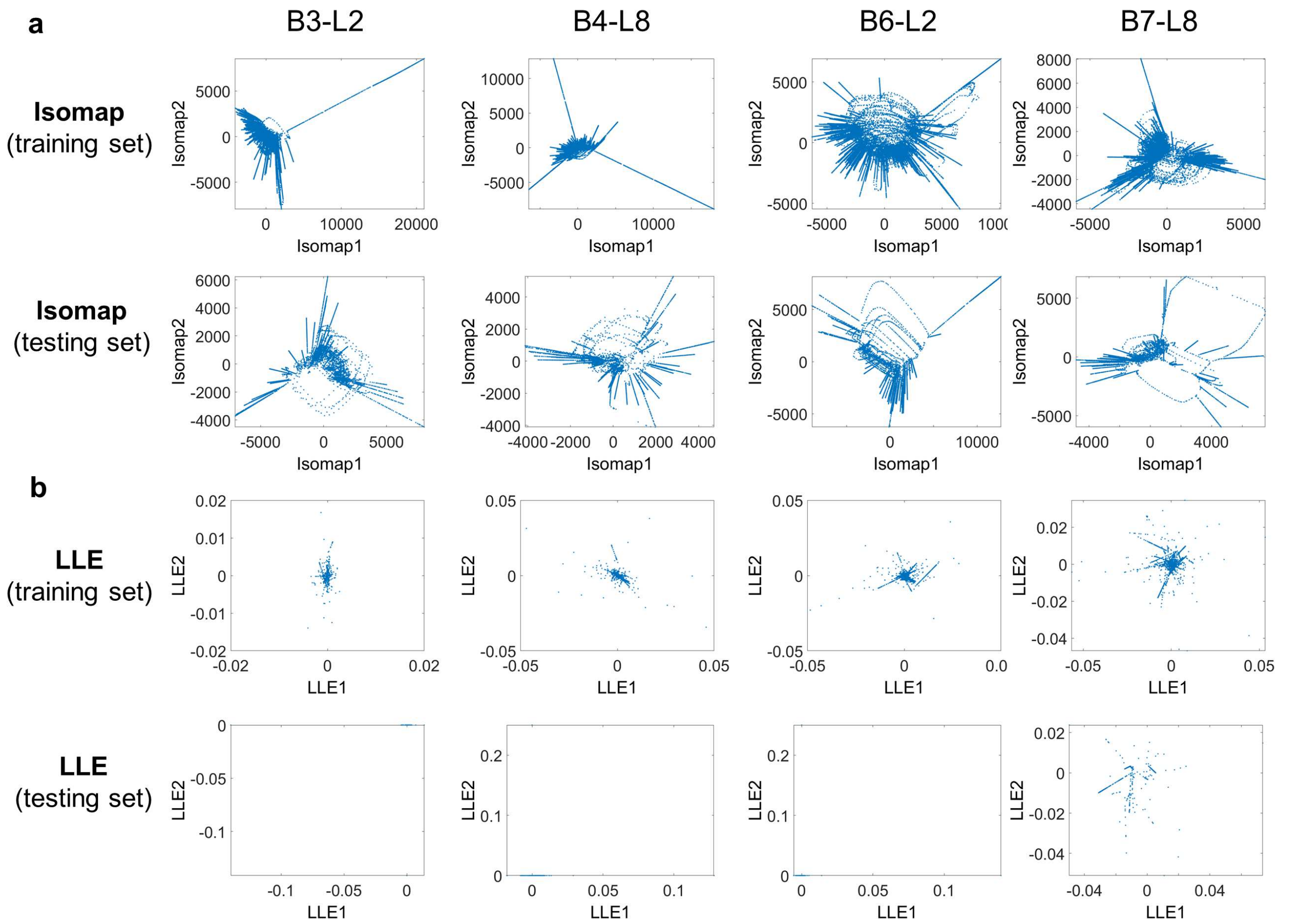

Fig. S26. Visualizations of the Dense-UNet features for segmentation task after training the network by (a) Isomap and (b) LLE. Here, B3-L2 denotes the 2nd layer of the 3rd dense block, B4-L8 denotes the 8th layer of the 4th dense block, B6-L2 denotes the 2nd layer of the 6th dense block, and B7-L8 denotes the last layer before the final output. Source data are provided as a Source Data file.

8. Quantification of the manifold structure

Quantifying the structure of a data manifold is a complex task, as manifolds can have intricate geometries and topologies. However, several techniques and metrics have been developed over the years to tackle this problem. Here are some ways to quantify the structure of a data manifold:

Intrinsic Dimensionality: This measures the minimum number of parameters needed to describe the data without much loss of information. There are various estimators like the Maximum Likelihood Estimator (MLE), the Two-NN estimator, and the Minimum Spanning Tree estimator. We use the Two-NN algorithm to estimate the intrinsic dimensionality of a given $m \times n$ matrix, where m is the number of data points and n is the number of features. Here's how the Two-NN algorithm works in a nutshell: 1) For each data point, find its two nearest neighbors. 2) Compute the distance r_1 to the nearest neighbor and r_2 to the second nearest neighbor. 3) Estimate the intrinsic dimensionality D using the formula:

$$D = \frac{1}{m} \sum_{i=1}^{m} \frac{\log\left(\frac{r_2(i)}{r_1(i)}\right)}{\log\left(\frac{1}{r_1(i)}\right)}$$

Curvature: The curvature of a manifold can give insights into its structure. There are different types of curvature measurements such as Gaussian curvature, mean curvature, and sectional curvature. High curvature regions can indicate "folds" or "bends" in the manifold. Let x_i be a point in the $m \times n$ dataset X, where m is the number of data points and n is the number of features (or dimensions). We consider the k nearest neighbors of x_i to form a local neighborhood. We then fit a local quadratic surface to this neighborhood. The equation of a general quadratic surface in n dimensions can be represented as:

$$f(x) = a_0 + a_1x_1 + a_2x_2 + \ldots + a_nx_n + a_{11}x_1^2 + a_{22}x_2^2 + \ldots + a_{nn}x_n^2 + 2a_{12}x_1x$$

After fitting such a surface to the local neighborhood of x_i, we can compute the shape operator S, which is a matrix that captures how the normal vector to the surface changes as we move along the surface. The eigenvalues $\lambda_1, \lambda_2, \ldots, \lambda_n$ of S give us the principal curvatures of the surface at x_i. Finally, the mean curvature H at x_i is defined as the average of these principal curvatures:

$$H = \frac{1}{n}\sum_{i=1}^{n}\lambda_i$$

Or, in terms of the principal curvatures $k_1, k_2, \ldots, k_n$:

$$H = \frac{1}{n}\sum_{i=1}^{n}k_i$$

Please note that these equations are a simplified and approximate way to estimate the mean curvature for a point cloud data set. The precise computation of mean curvature usually involves differential geometry and may require solving partial differential equations, especially for irregularly sampled or high-dimensional data.

Geodesic Distance: The shortest path between two points on a manifold is called a geodesic. Computing geodesic distances can provide insights into how data points are interconnected within the manifold. Computation of geodesic distance has been discussed in detail in the main manuscript.

Performance comparison in preserving manifold structure. In Table S3, performance improvement is computed by $100 \times (P_s - P_g)/P_s$, where P_g and P_s are the absolute error (AE) from our calculation and LLE, respectively. P denotes the AE between the manifold quantification index computed from HD feature data and low dimensional representation from MDA and LLE.

Performance comparison in follow up tasks. We have added evaluations of MDA and existing methods on follow up tasks in Table S4. In Table S4, performance improvement for regression tasks is computed by $100 \times (P_s - P_g)/P_s$, where P_g are P_s are the root mean square (RMSE) of our calculation and the best performing existing method. For classification tasks, performance improvement is computed by $100 \times (P_g - P_s)/P_s$, where P_g are P_s are the classification accuracy of our calculation and the best performing existing method, respectively. Below we describe the computational process of the indices in follow up tasks.

Root mean square error: For tasks involving image segmentation, superresolution, and gene expression prediction, we first compute the geodesic distance (8) among data points based on the HD labels over the manifold. We find one end point of the manifold (see Fig. 1) and the distance from that point to others is used as labels in the follow up regression task. We allocate 70% of the data randomly to train a k-NN regressor (with k=5), aiming to predict the distance from the low-dimensional representations produced by various methodologies, and reserve the remaining 30% for testing. Similarly, for angle and survival prediction tasks, we use 70% of the data to train a k-NN regressor (with k=5) for predicting angles and survival durations from different low-dimensional method representations, with the remaining 30% designated for testing. We used root mean square error (RMSE) between the actual value and predicted value to train the models. Performance metrics are consistently provided as the mean of a 5-fold cross-validation.

Classification accuracy: Following common practice in machine learning community, we chose 70% of the data for training a k-NN classifier (with k=5) and 30% for testing. The mean performance for 5-fold cross validation is reported.

Table S3. Performance comparison of MDA with LLE in preserving manifold structure information

Dataset	Task	Performance metric	Performance improvement by MDA	Results
TCGA	Survival prediction	Geodesic	18%	Figs. 5, S27
		Intrinsic dimensionality	11%	Figs. 5, S27
		Mean curvature	15%	Figs. 5, S27
L1000	Gene expression change prediction	Geodesic distance	39%	Fig. 5
BraTS	Image segmentation	Geodesic distance	14%	Fig. 3
ISIC-2019	Super resolution	Geodesic distance	12%	Fig. 6
MNIST	Angle prediction	Geodesic distance	25%	Fig. E3, S27
		Intrinsic dimensionality	21%	Fig. E3, S27
		Mean curvature	26%	Fig. E3, S27

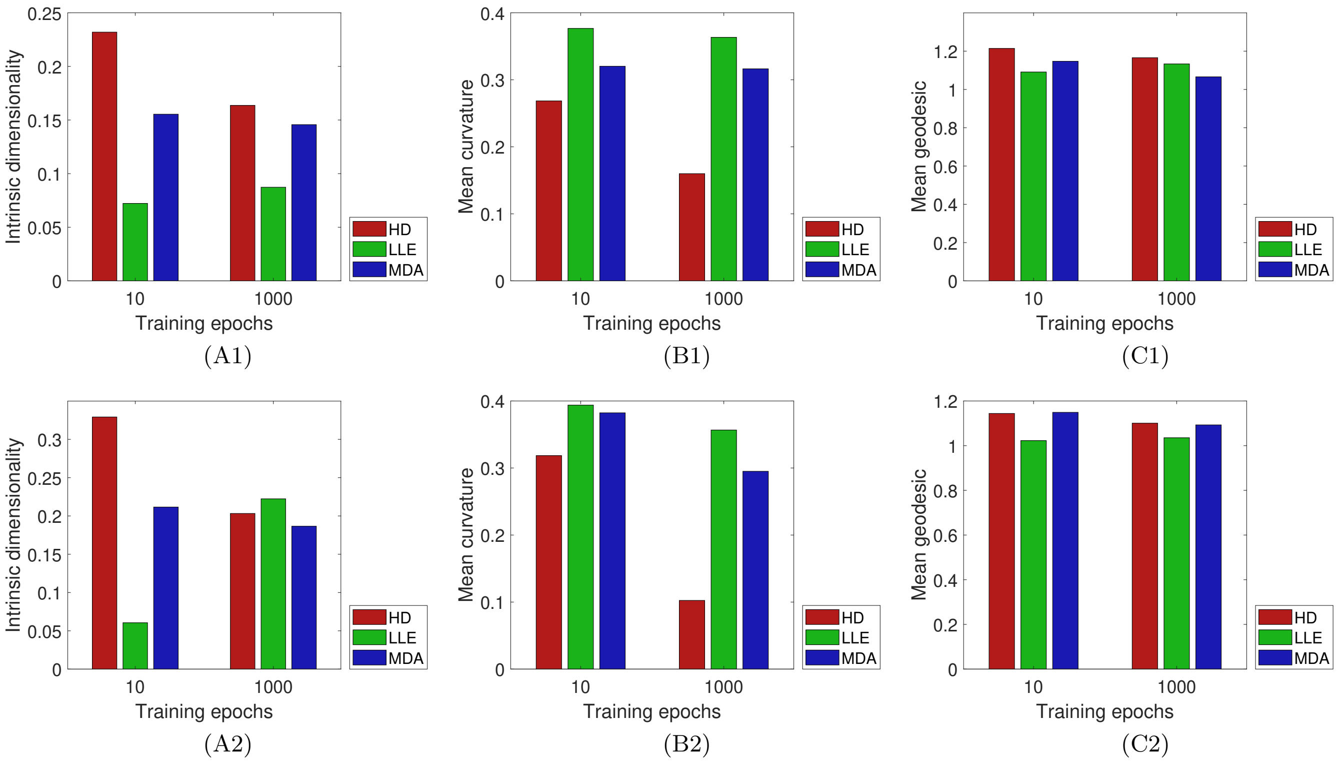

Fig. S27. Quantification of manifold structure using (A) intrinsic dimensionality, (B) mean curvature, and (C) mean geodesic distance for last fully connected layer of mCNN ((Table S6)) and Dense-UNet networks trained on MNIST (1) and BraTS image segmentation (2) datasets. For MNIST dataset, the image number is 5000 (n=5000) and for BRaTS segmentation dataset, the number of images is 20000 (n=20000). The indices are computed from HD data and low dimensional representations from MDA and LLE methods. It is seen from indices computed from HD data and MDA results that as training progresses, the manifold geometry becomes smooth and mean curvature, intrinsic dimensionality and geodesic reduces. LLE fails to show such insight. Source data are provided as a Source Data file.

Table S4. Performance comparison of MDA with t-SNE, UMAP, Isomap and LLE for follow up tasks

**Dataset**	**Data type**	**Task**	**Performance index**	**Improvement by MDA**	**Results**
BraTS	Image	Image segmentation	RMSE	14%	Fig. 3
ISIC-2019	Image	Super resolution	RMSE	12%	Fig. 6
COVID	Image	Classification	kNN-Accuracy	11%	Fig. 7
Diabetic retinopathy	Image	Classification	kNN-Accuracy	18%	Fig. S41
MNIST	Image	Angle prediction	RMSE	25%	Fig. E3
TCGA	Tabular	Survival prediction	RMSE	28%	Fig. 4
L1000	Tabular	Gene expression prediction	RMSE	39%	Fig. 5

9. Neural collapse in regression tasks

Neural collapse is an extreme phenomenon when cross-example within-class variability of the last-layer training features (activations) approaches zero in deep learning classification tasks. However, this phenomenon happens only for the features of the last fully connected layers (FCLs) in the extreme case during the terminal phase of deep learning training (9–13). Note that the terminal phase of training (TPT) begins at the epoch where the training error starts vanishing. During TPT, the training error stays effectively zero, while the training loss is pushed toward zero. For regression problems, neural collapse phenomenon is not yet obvious. Below, for two datasets (TCGA and MNIST), we train two networks (mCNN and fMLP-see Tables S5 and S6) for 10000 epochs and the last FCL is visualized by MDA and t-SNE. The results are added in Figs. S28 and S29 for TCGA and MNIST datasets, respectively. For both datasets, it is observed from MDA visualizations that at 10000 epochs (neural collapse), the feature manifold becomes simplified curves. t-SNE fails to show such insights.

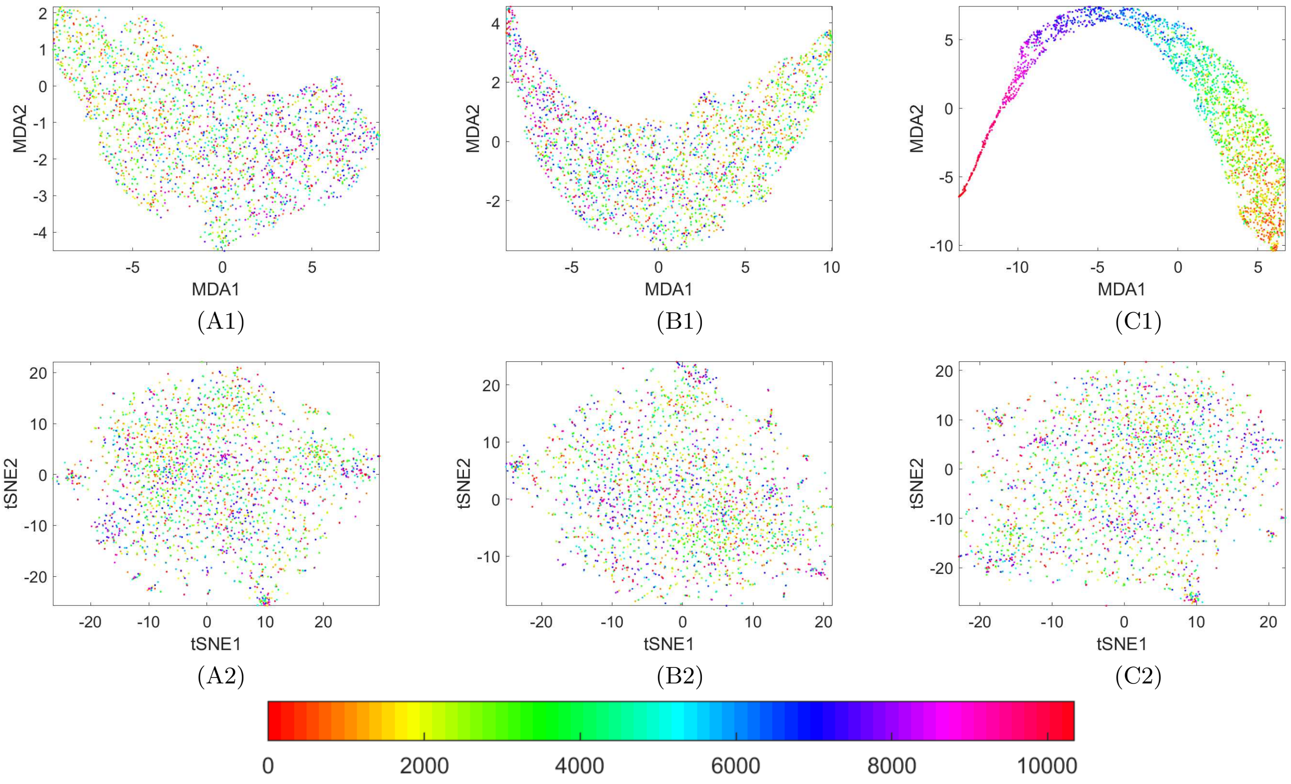

Fig. S28. MDA (1) and t-SNE (2) visualizations of training data features from last FCL of fMLP trained on TCGA dataset at epoch of 1 (A), 20 (B) and 10000 (C). MDA visualization shows that the features lie on a simplified manifold when the network is trained for 10000 epochs (neural collapse). t-SNE fails to show such interesting observation. The colorbar denotes the patient survival in days. Source data are provided as a Source Data file.

Table S5. Layer configurations of mCNN

Layer type	Activations	Learnables
Image Input	$28 \times 28 \times 1 \times 1$	–
Convolution	$28 \times 28 \times 8 \times 1$	Weights $3 \times 3 \times 1 \times 8$, Bias $1 \times 1 \times 8$
Batch normalization	$28 \times 28 \times 8 \times 1$	Offset $1 \times 1 \times 8$, Scale $1 \times 1 \times 8$
ReLU	$28 \times 28 \times 8 \times 1$	–
Average pooling	$14 \times 14 \times 8 \times 1$	–
Convolution	$14 \times 14 \times 16 \times 1$	Weights $3 \times 3 \times 8 \times 16$, Bias $1 \times 1 \times 16$
Batch normalization	$14 \times 14 \times 16 \times 1$	Offset $1 \times 1 \times 16$, Scale $1 \times 1 \times 16$
ReLU	$14 \times 14 \times 16 \times 1$	–
Average pooling	$7 \times 7 \times 16 \times 1$	–
Convolution	$7 \times 7 \times 32 \times 1$	Weights $3 \times 3 \times 16 \times 32$, Bias $1 \times 1 \times 32$
Batch normalization	$7 \times 7 \times 32 \times 1$	Offset $1 \times 1 \times 32$, Scale $1 \times 1 \times 32$
ReLU	$7 \times 7 \times 32 \times 1$	–
Dropout	$7 \times 7 \times 32 \times 1$	–
Convolution	$7 \times 7 \times 32 \times 1$	Weights $3 \times 3 \times 32 \times 32$, Bias $1 \times 1 \times 32$
Batch normalization	$7 \times 7 \times 32 \times 1$	Offset $1 \times 1 \times 32$, Scale $1 \times 1 \times 32$
ReLU	$7 \times 7 \times 32 \times 1$	–
Dropout	$7 \times 7 \times 32 \times 1$	–
Fully Connected	$1 \times 1 \times 1 \times 1$	Weights 1×1568, Bias 1×1
Regression layer	$1 \times 1 \times 1 \times 1$	-

Table S6. Layer configurations of fMLP

Layer type	Activations	Learnables
Feature Input	$20,000 \times 1$	–
Fully Connected	1024×1	Weights 1024×20000, Bias 1024×1
ReLU	1024×1	–
Fully Connected	512×1	Weights 512×1024, Bias 512×1
ReLU	512×1	–
Fully Connected	256×1	Weights 256×512, Bias 256×1
Batch Normalization	256×1	–
Dropout	256×1	–
Fully Connected	128×1	Weights 128×256, Bias 128×1
ReLU	128×1	–
Fully Connected	64×1	Weights 64×128, Bias 64×1
Fully Connected	1×1	Weights 1×64, Bias 1×1
Regression layer	1×1	-

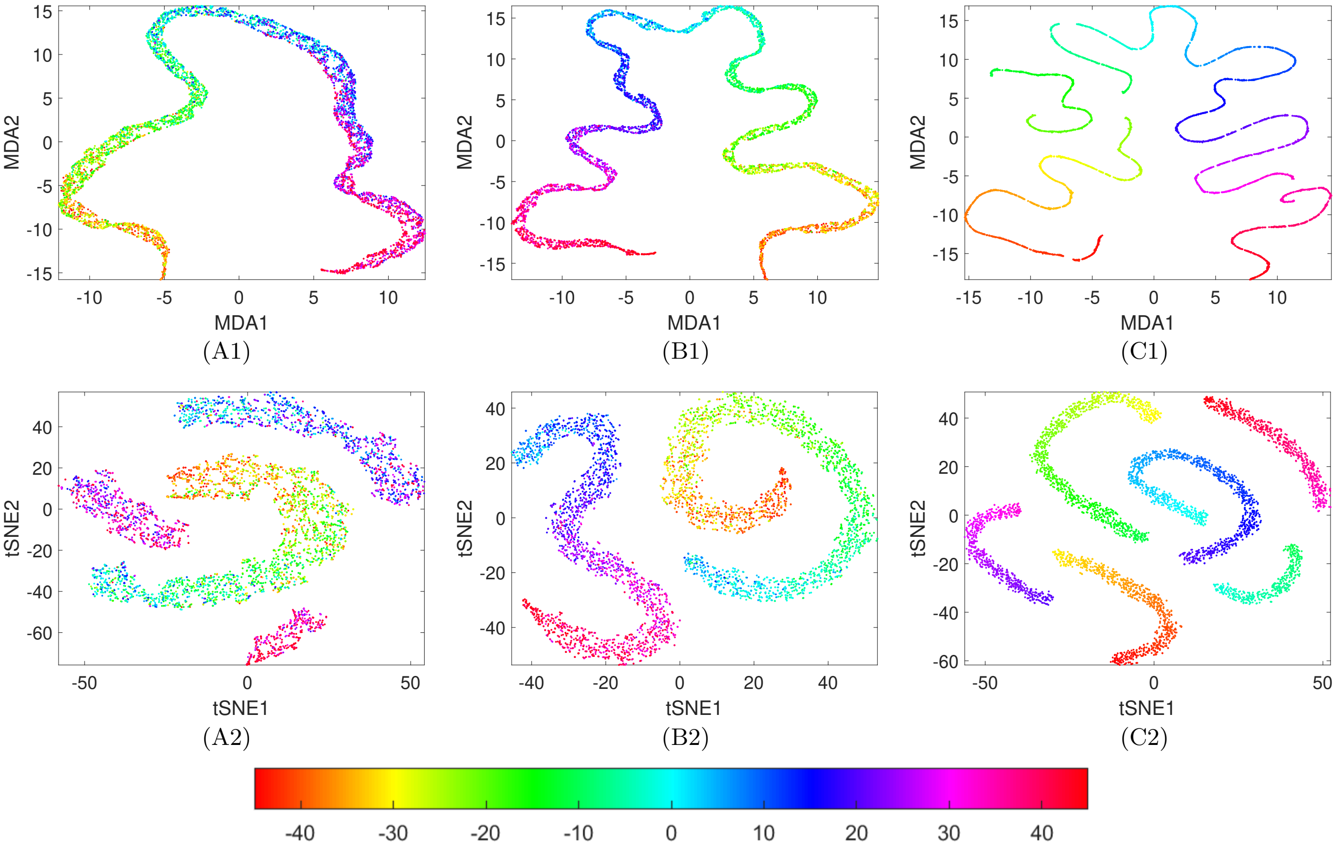

Fig. S29. MDA (1) and t-SNE (2) visualizations of training data features from last FCL of mCNN trained on MNIST dataset at epoch of 10 (A), 20 (B) and 10000 (C). MDA visualization shows that the features lie on a simplified manifold when the network is trained for 10000 epochs (neural collapse). The colorbar denotes digit angles in degrees. Source data are provided as a Source Data file.

10. Effect of different layers on DNN feature space

To analyze the effect of different layers on the DNN feature space, we chose mCNN and fMLP (see Tables S5 and S6) with TCGA and MNIST datasets. We show the MDA and t-SNE visualizations of the different layers (RELU, batch normalization, and dropout) in Figs. S30, S31, S32 and S33 for two different datasets (MNIST and TCGA). It is evident from MDA visualizations that RELU and batch normalization layers smoothen the manifold continuity and correct the distance of the data points over the manifold. In other words, these layers help the network learn the relationship between the data and the label. The dropout layer is known to have no impact on the feature space other than making the features sparse. Similar MDA visualizations of the features before and after dropout layers confirm this observation. Such insights can not be acquired from t-SNE visualizations.

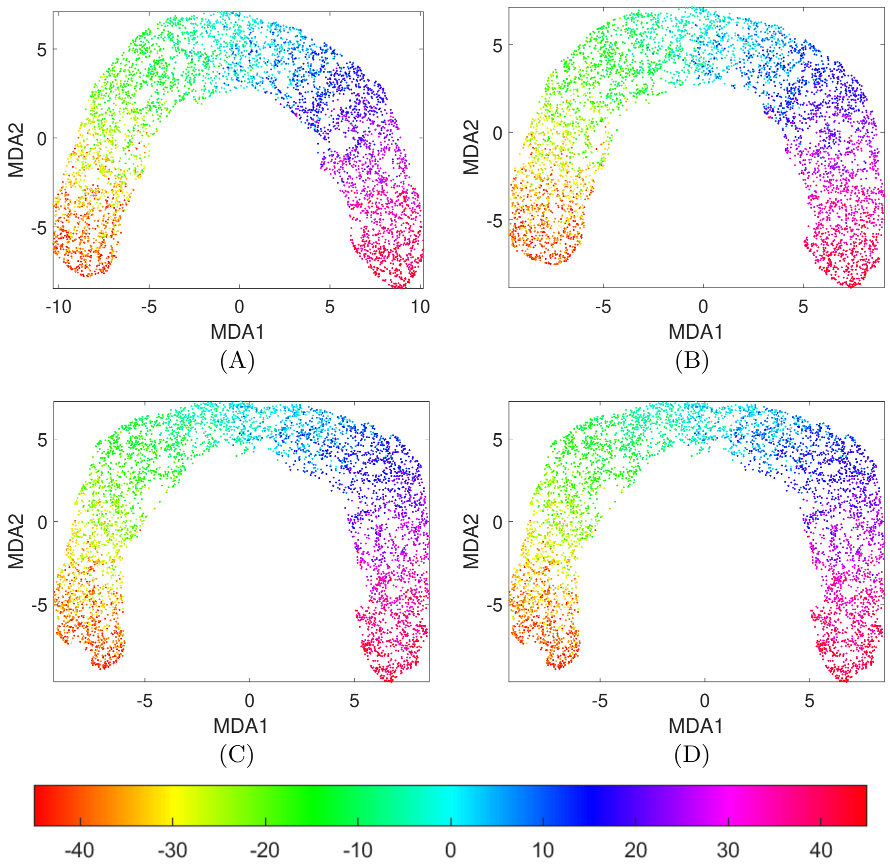

Fig. S30. Effects of different layers on mCNN features extracted from MNIST dataset for angle prediction task. MDA visualized features after (A) convolutional layer, (B) batch normalization layer, (C) RELU layer and (D) dropout layer. It is seen that batch normalization and RELU layers attempt to smooth the features, whereas dropout layer has no effect. The colorbar denotes digit angles. Source data are provided as a Source Data file.

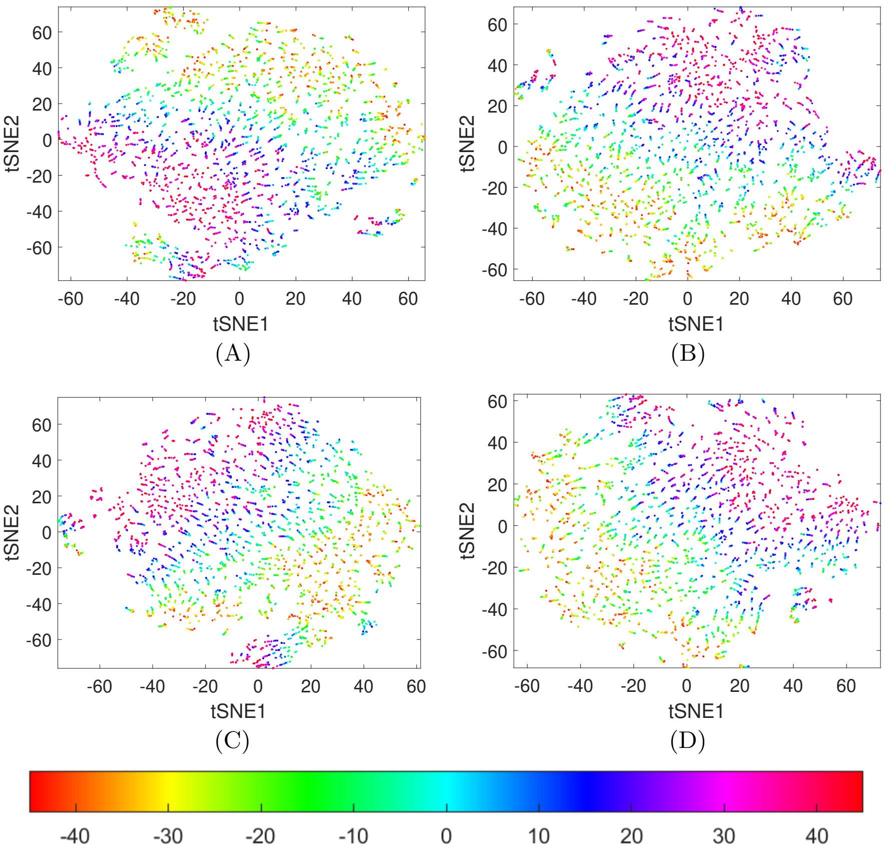

Fig. S31. Effects of different layers on mCNN features extracted from MNIST dataset for angle prediction task. t-SNE Visualized features after (A) convolutional layer, (B) batch normalization layer, (C) RELU layer and (D) dropout layer. The colorbar denotes digit angles. Source data are provided as a Source Data file.

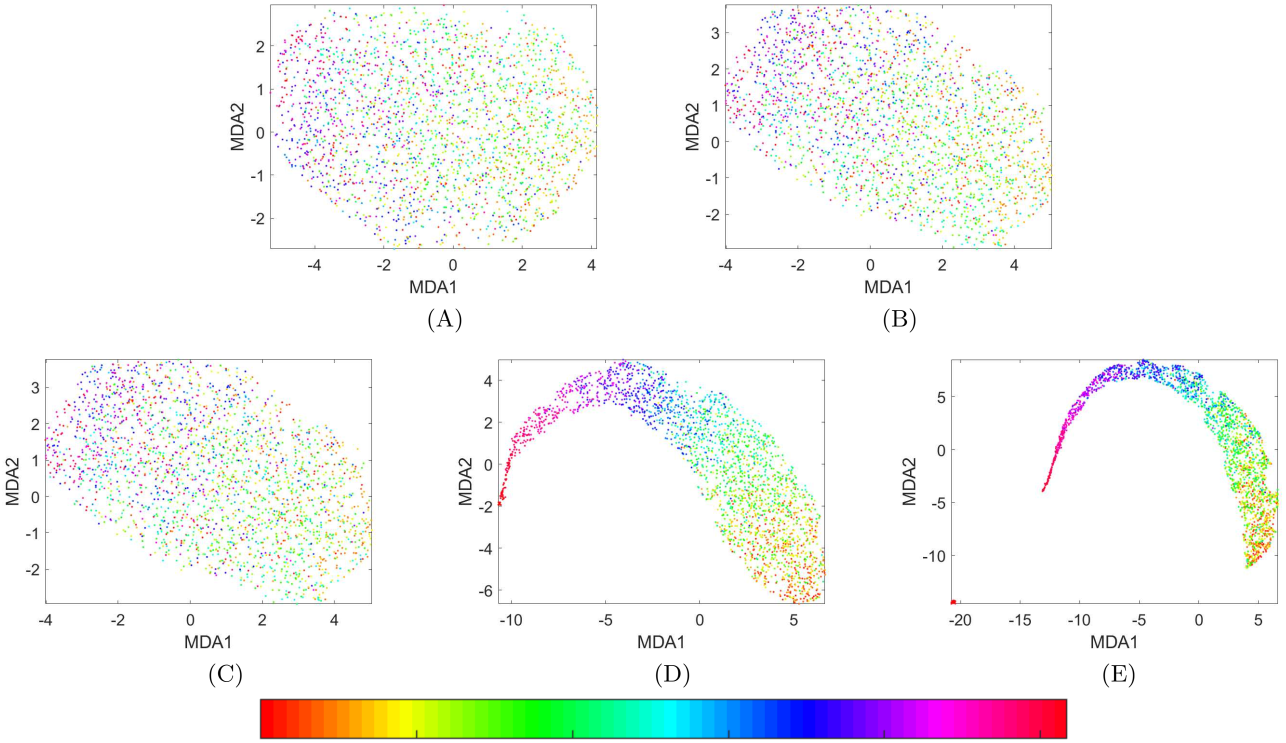

Fig. S32. Effects of different layers on fMLP features extracted from TCGA dataset for survival prediction task. Visualized features after (A) FCL layer, (B) batch normalization layer, (C) dropout layer, (D) FCL layer and (E) RELU layer. It is seen that batch normalization and RELU layers attempt to smooth the features, whereas dropout layer has no effect. The colorbar denotes the patient survival days. Source data are provided as a Source Data file.

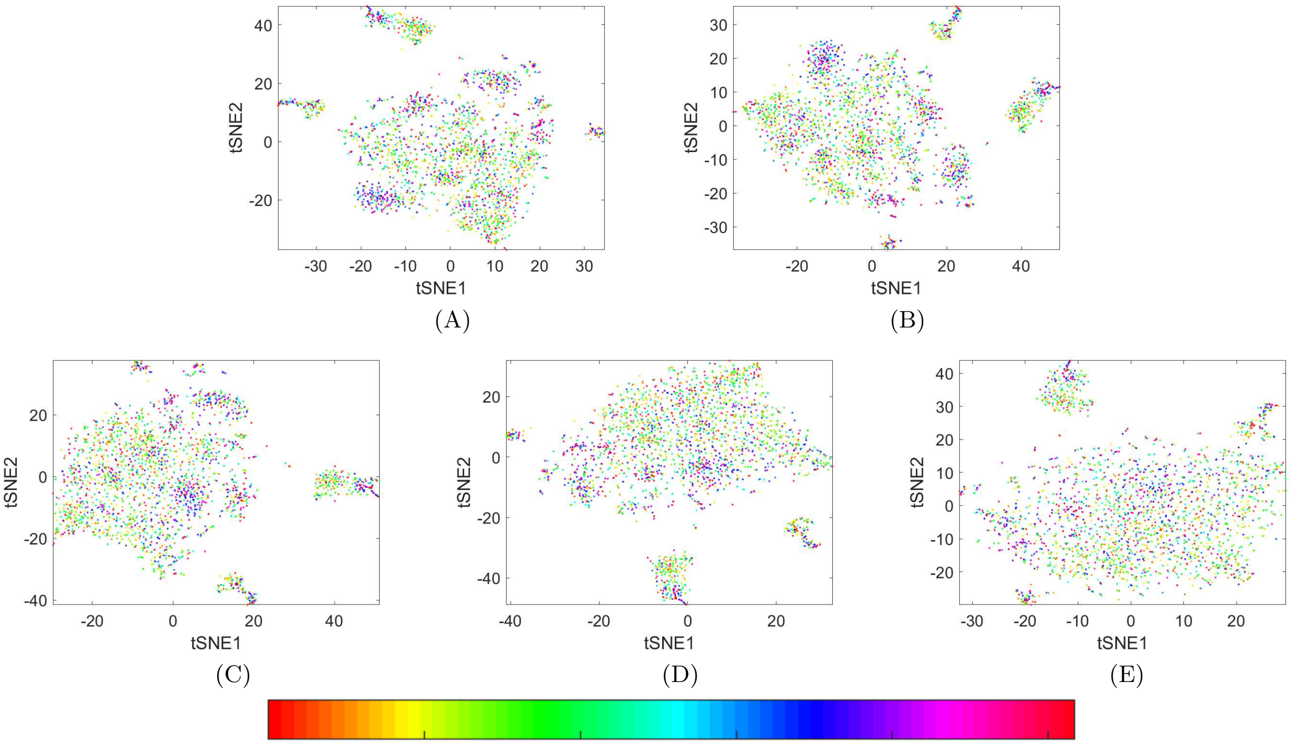

Fig. S33. Effects of different layers on fMLP features extracted from TCGA dataset for survival prediction task. t-SNE Visualized features after (A) FCL layer, (B) batch normalization layer, (C) dropout layer, (D) FCL layer and (E) RELU layer. The colorbar denotes the patient survival days. Source data are provided as a Source Data file.

11. Analysis of DNN features for extrapolation tasks

For analyzing the DNN feature space in extrapolation tasks, we chose mCNN and fMLP (see Tables S5 and S6) with TCGA and MNIST datasets. We show the MDA and t-SNE visualizations of the different layers in Figs. S34, S35, S36 and S37. In these figures, we provide MDA visualizations for two networks trained with partial labels on TCGA and MNIST datasets. In the former case, we selected TCGA data with patients with survival days ranging from 0 to 7000 days. We trained the network with this data and tested it on patient data with survival days ranging from 0 to 10000 days. MDA visualization reveals that the network projects the data of higher angles ($>$ 7000 days) between 5000 to 10000 days. The network identifies similar patient data in this unknown day range from the training data and projects the data to a similar position as the training data point. Similar insights can be obtained from MDA visualizations for the MNIST dataset. The DNN is trained on MNIST data with -45-20 degree angle data and tested with data of -45-45 degree angle.

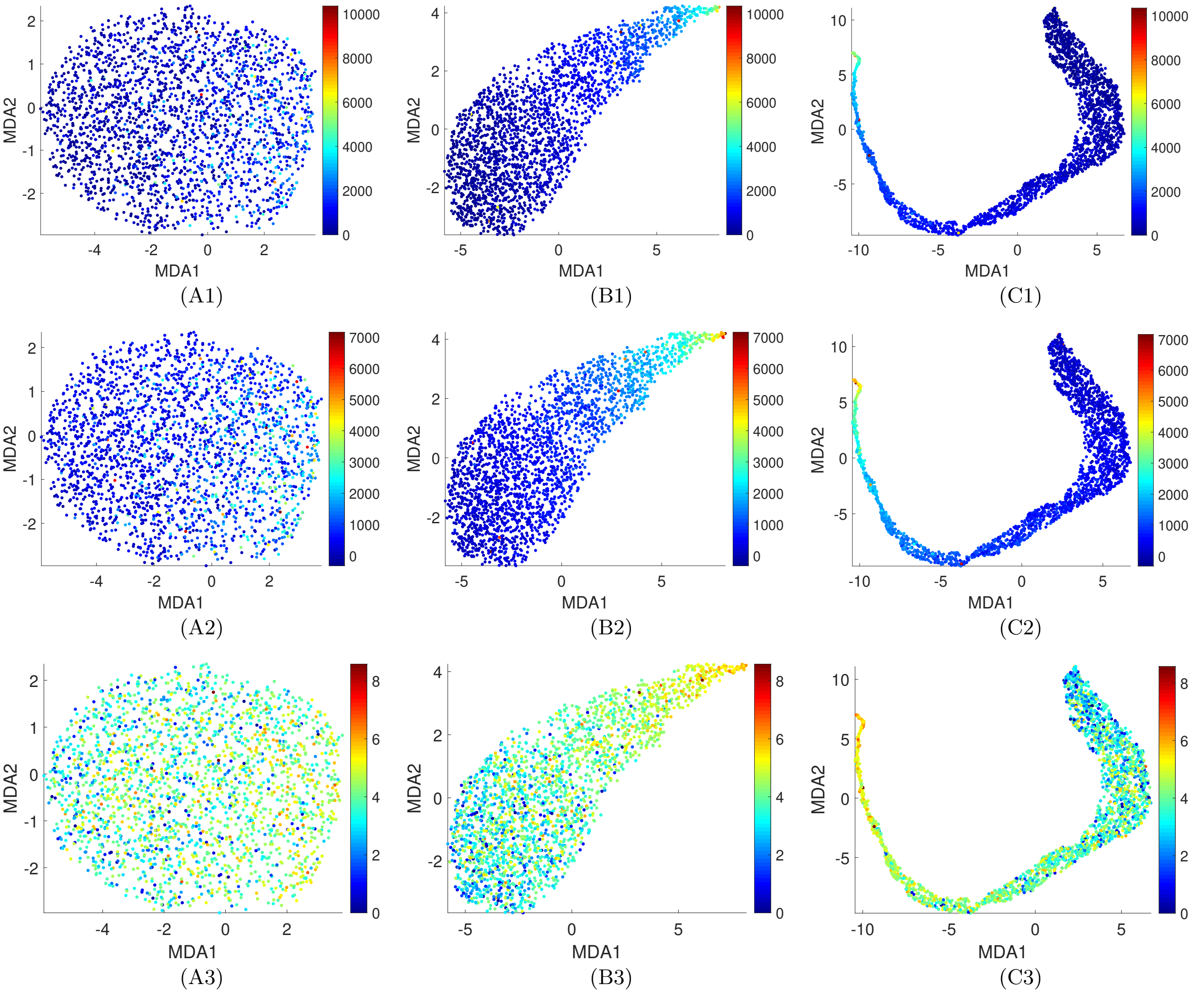

Fig. S34. MDA Visualizations of the feature space when the fMLP is trained on TCGA data with 0-7000 days survival data and tested with data of 0-10000 days survival data. Columns A, B and C denote the layers 9, 10 and 11 of the fMLP, respectively. The colormaps correspond to (row 1) test labels, (row 2) predicted labels and (row 3) absolute prediction error $\log(|Ypred - Ytest|)$. Source data are provided as a Source Data file.

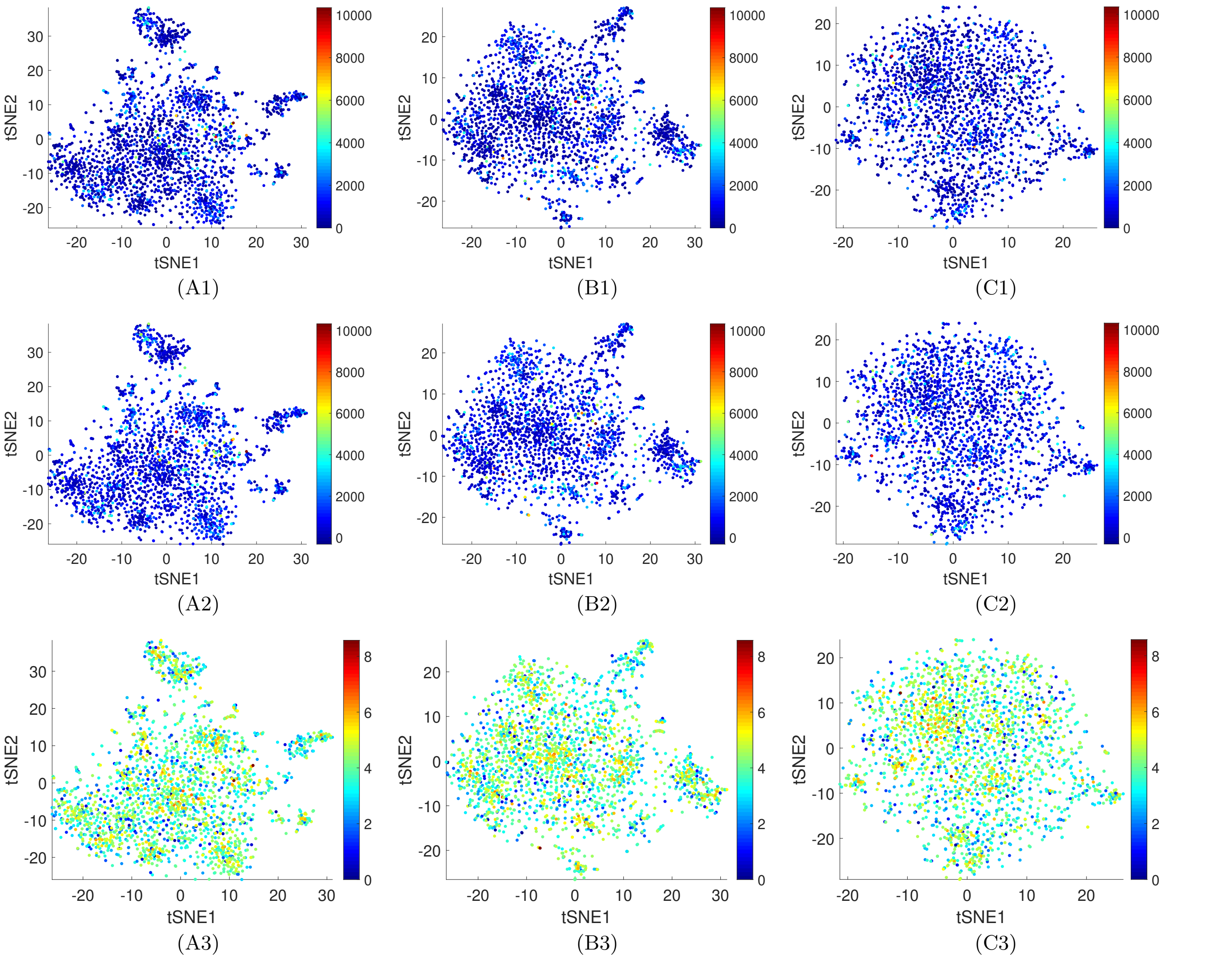

Fig. S35. t-SNE Visualizations of the feature space when the DNN is trained on TCGA data with 0-7000 days survival data and tested with data of 0-10000 days survival data. Columns A, B and C denote the layers 9, 10 and 11 of the fMLP, respectively. The colormaps correspond to (row 1) test labels, (row 2) predicted labels and (row 3) absolute prediction error $\log(|Ypred - Ytest|)$. Source data are provided as a Source Data file.

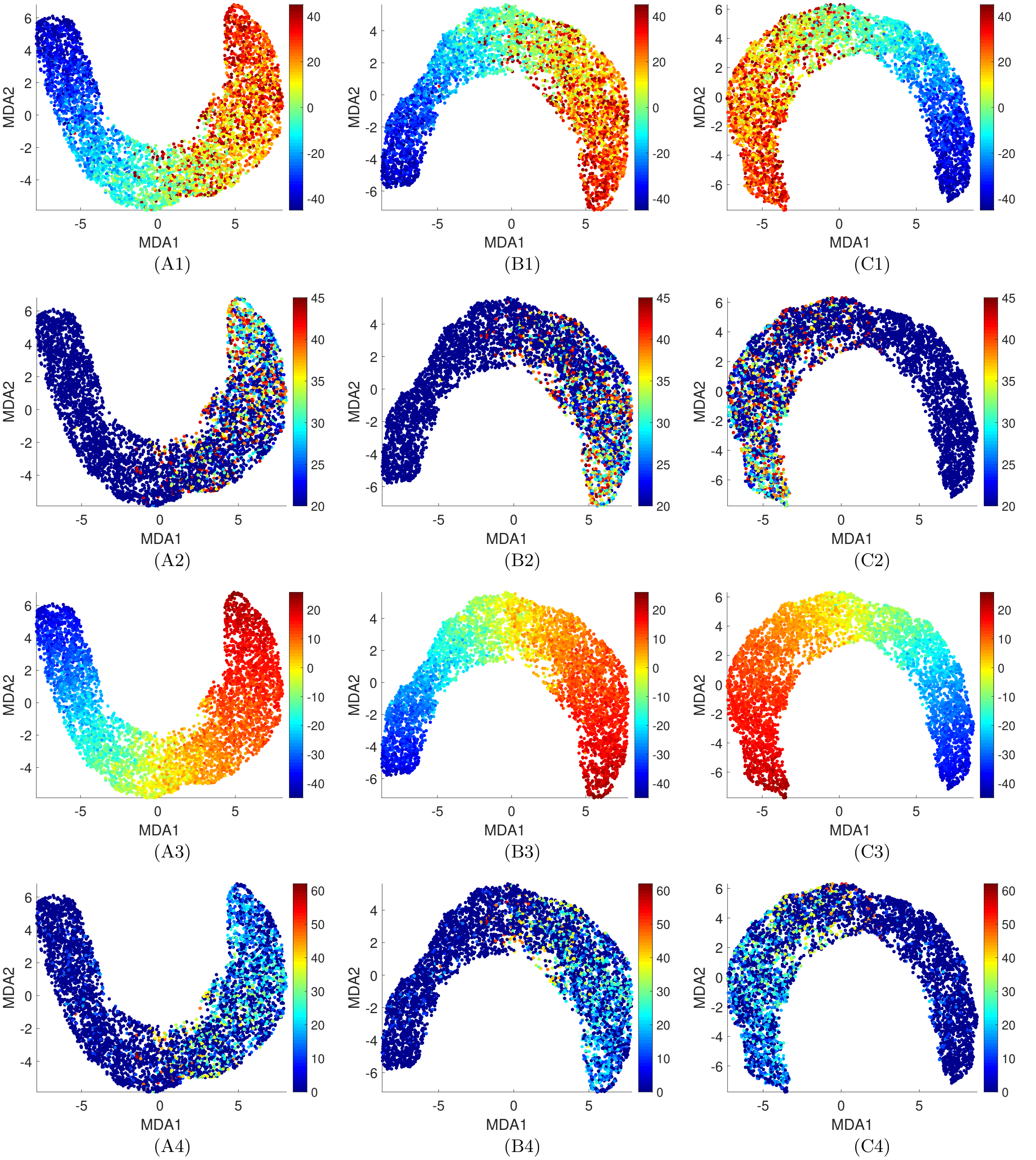

Fig. S36. MDA Visualizations of the feature space when the DNN is trained on MNIST data with -45-20 degree angle data and tested with data of -45-45 degree angle. Columns A, B and C denote the layers 14, 15 and 17 of the mCNN, respectively. The colormaps correspond to (row 1) test labels (row 2) test labels with colormap representing 20-45 degree angles only, (row 3) predicted labels and (row 4) absolute prediction error (|Ypred-Ytest|). For second row, all the values between -45-20 are set to blue to better track the projected points between 20-45 degree. Source data are provided as a Source Data file.

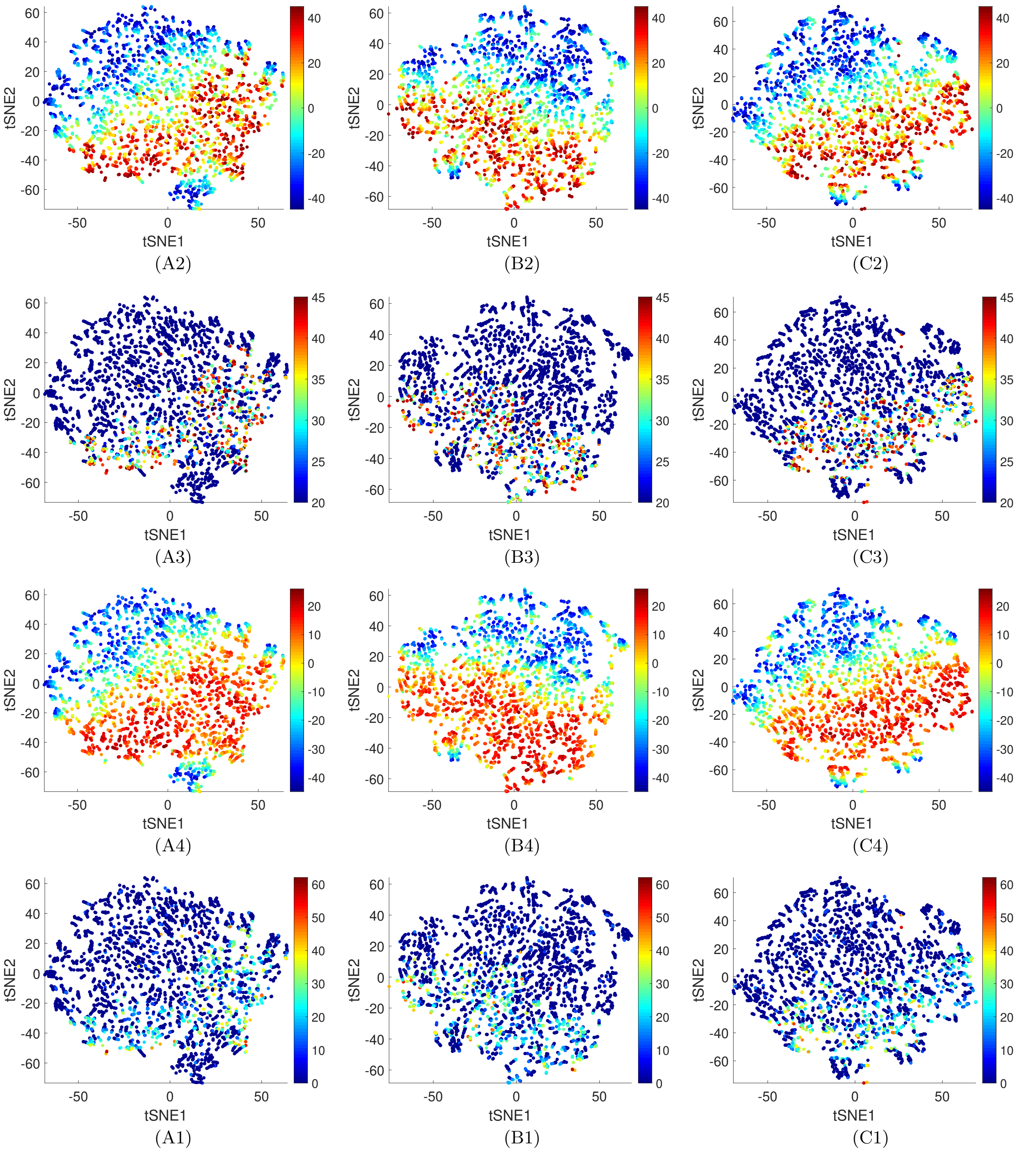

Fig. S37. t-SNE Visualizations of the feature space when the DNN is trained on MNIST data with -45-20 degree angle data and tested with data of -45-45 degree angle. Columns A, B and C denote the layers 14, 15 and 17 of the mCNN, respectively. The colormaps correspond to (row 1) test labels (row 2) test labels with colormap representing 20-45 degree angles only, (row 3) predicted labels and (row 4) absolute prediction error (|Ypred-Ytest|). For second row, all the values between -45-20 are set to blue to better track the projected points between 20-45 degree. Source data are provided as a Source Data file.

12. Effect of different hyperparameters on MDA visualizations

The hyper-parameters of the prior gamma distribution can be finetuned via cross-validation to improve the performance of the dimensionality reduction in MDA. Specifically, one could compute different projection matrices $\mathbf{q}(\mathbf{Q})$ via a grid-search on the scaling parameters of the gamma distribution, and subsequently evaluate the performance of the resulting projection matrices. Nevertheless, in practice, we have observed a good performance with the set of parameters we have selected. As the reviewer has alluded, the Bayesian dimensionality reduction algorithm is insensitive to these parameters, and it performs well across a wide range of hyperparameters for prior gamma distribution.

In the revised manuscript, we have indeed performed a grid search over these parameters for each dataset to demonstrate that the selected hyper-parameters in the manuscript are good values for the Bayesian dimensionality reduction algorithm. The results of the grid search are added in Figs. S39 and S38.

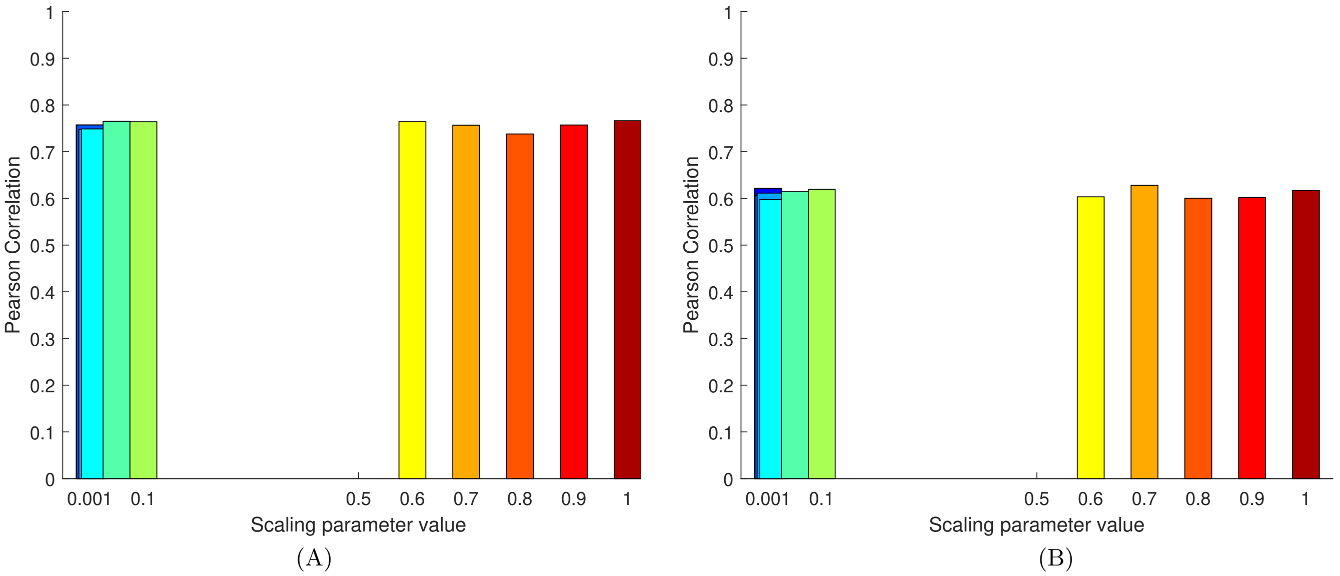

Fig. S38. Pearson correlation between distances among data points in low dimensional representation and HD feature data from fMLP and SRGAN network for grid search over different values of scaling parameters for (A) TCGA and (B) ISIC-2019 datasets. $\alpha_\phi = \beta_\phi = \alpha_\lambda = \beta_\lambda = \alpha_\psi = \beta_\psi$ is used for the experiments. Source data are provided as a Source Data file.

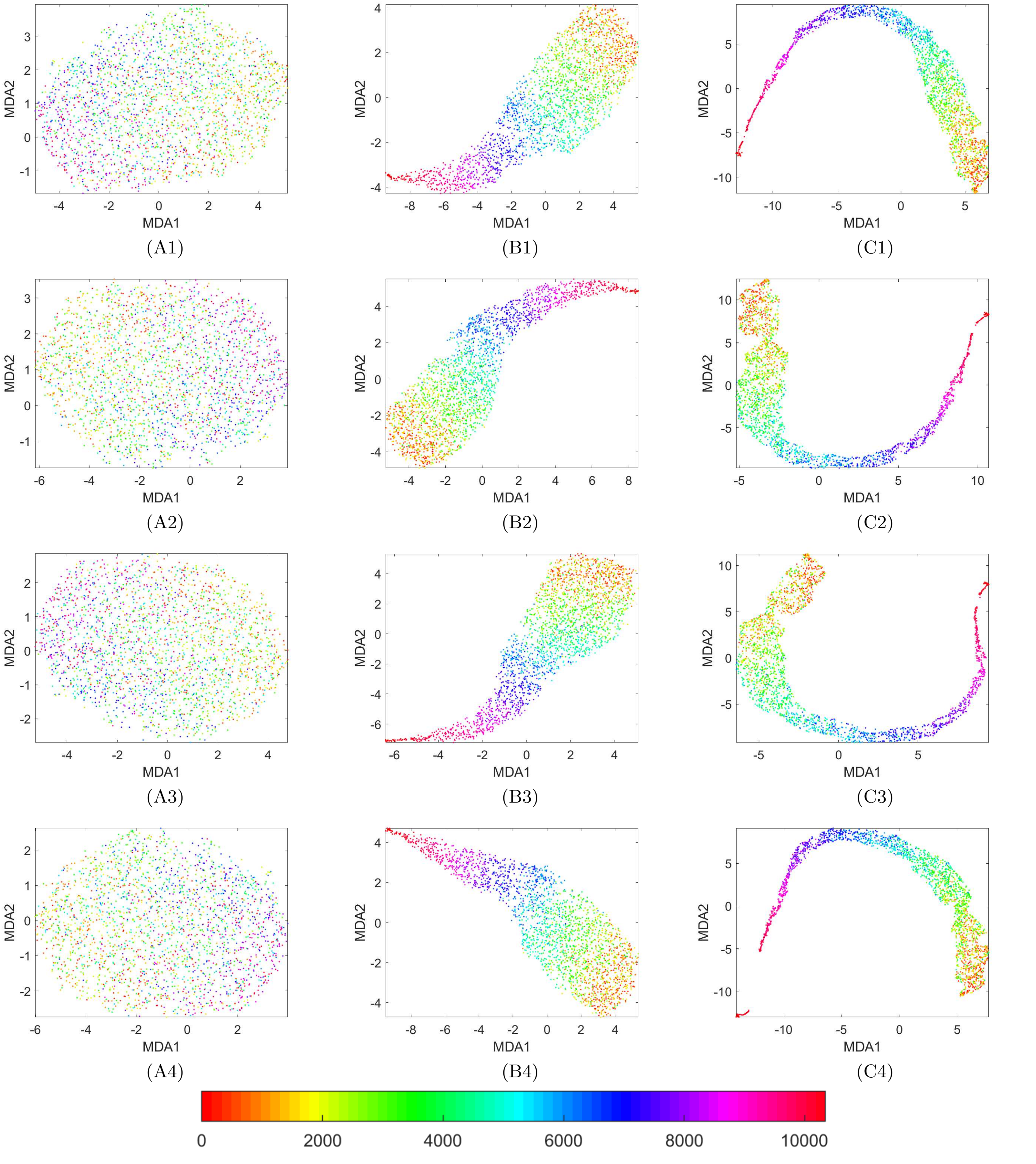

Fig. S39. Effect of different hyperparameters on MDA visualizations. MDA visualizations of layers of 6(A), 7(B), 8(C) from fMLP network (trained on TCGA dataset) with scaling parameter 1(1), 0.1(2), 0.001(3) and randomly chosen value between 0 and 1(4). $\alpha_\phi = \beta_\phi = \alpha_\lambda = \beta_\lambda = \alpha_\psi = \beta_\psi$ is used for the experiments. The colorbar denotes the patient survival days. Source data are provided as a Source Data file.

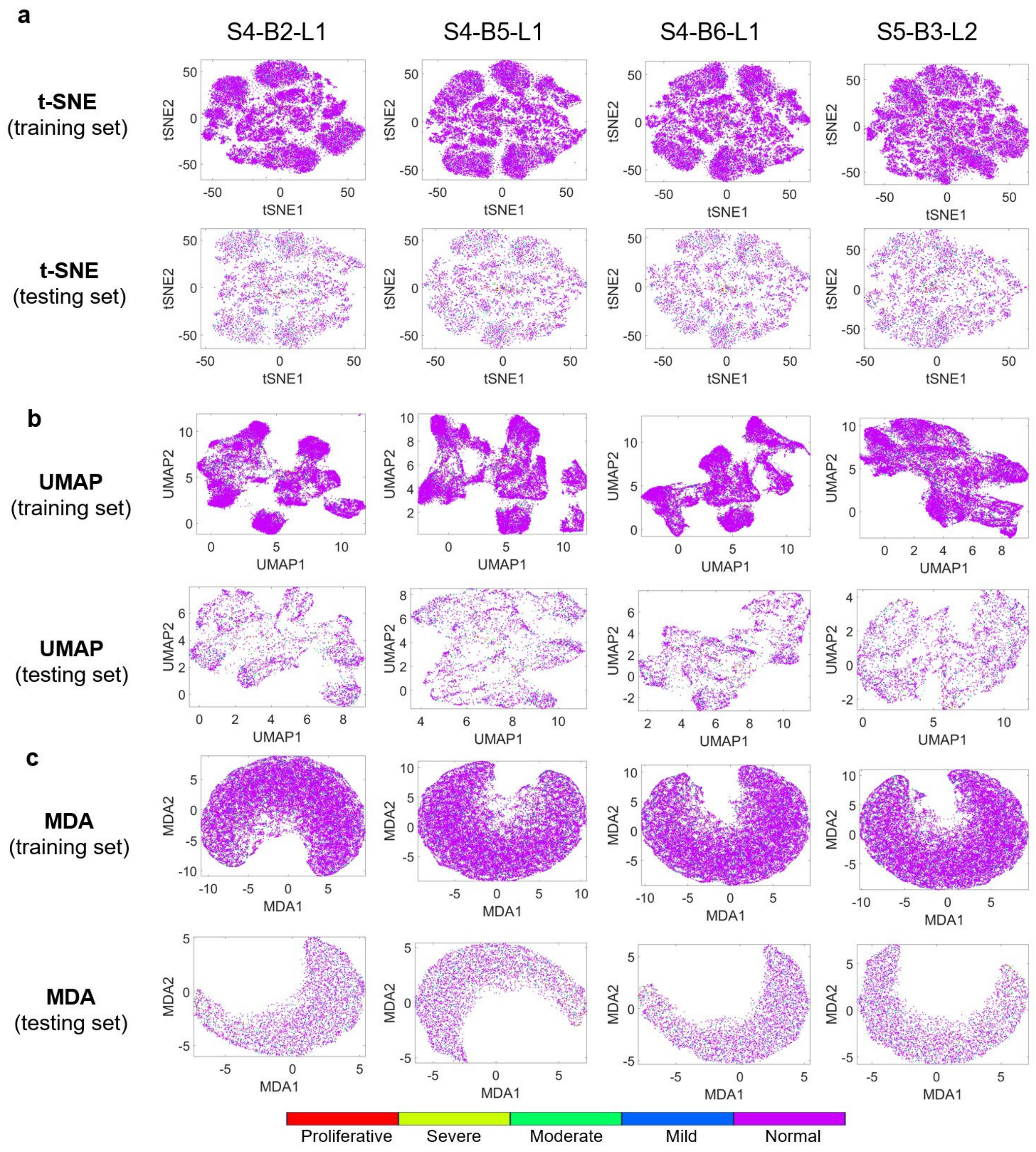

Fig. S40. Investigation of the feature space of ResNet50 network applied on a public DR dataset for classification into four categories. (a, b, c) t-SNE, UMAP, and MDA visualizations of the feature spaces at four different layers before training. Here, S2-B4-L3 denotes the 4th residual block's last convolutional layer in substructure 2, S3-B2-L3 denotes the 2nd residual block's last convolutional layer in substructure 3, S3-B6-L3 denotes the 6th residual block's last convolutional layer in substructure 3, and S4-B3-L3 denotes the 3rd residual block's last convolutional layer in substructure 4. Before training, the data points are randomly distributed in MDA visualizations. Source data are provided as a Source Data file.

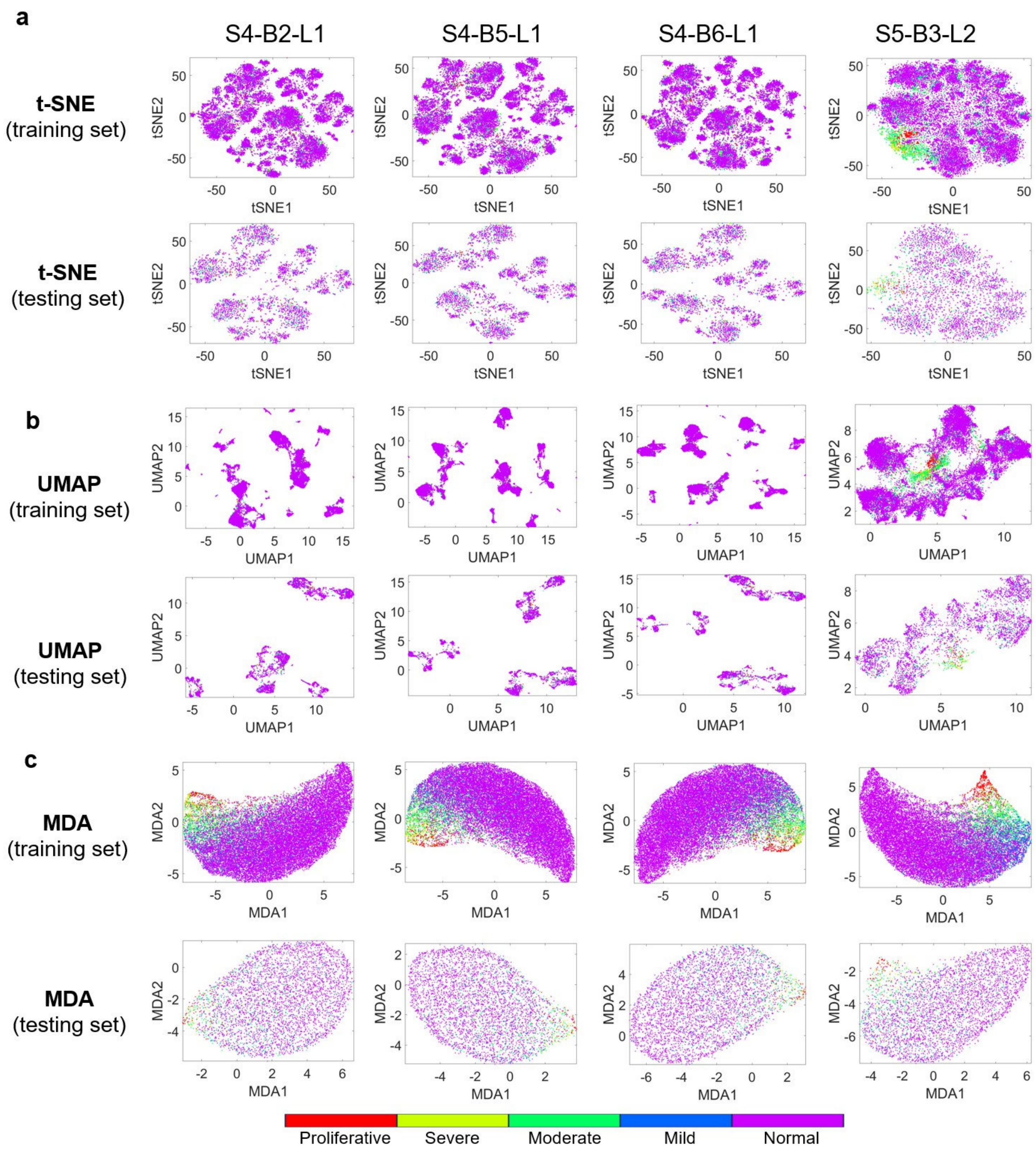

Fig. S41. Investigation of the feature space of ResNet50 network applied on a public DR dataset for classification into four categories. (a, b, c) t-SNE, UMAP, and MDA visualizations of the feature spaces at four different layers after training. Here, S2-B4-L3 denotes the 4th residual block's last convolutional layer in substructure 2, S3-B2-L3 denotes the 2nd residual block's last convolutional layer in substructure 3, S3-B6-L3 denotes the 6th residual block's last convolutional layer in substructure 3, and S4-B3-L3 denotes the 3rd residual block's last convolutional layer in substructure 4. Before training, the data points are randomly distributed in MDA visualizations. However, after the training, the feature space becomes well clustered in MDA visualizations, especially in deeper layers. t-SNE and UMAP fail to show any information about the training status of the network. The MDA results show that the features display a continuous distribution in the manifold space as the condition worsens (from normal to proliferative). Source data are provided as a Source Data file.

13. Effect of noise on DNN feature space

Below, we added results of robustness experiments for multiple noise levels and two different networks to show how MDA visualizations can be used to find a more robust network. From Fig. S42, it is obvious that the MDA visualizations of the robust Dense-UNet features show better continuous distribution of colors in comparison to MDA visualizations of features from less robust simple U-Net (14) (Fig. S43) at the same SNR levels. The segmentation performance in terms of Dice scores also support the conclusion. For quantitative analysis, we have added the Pearson correlation between the MDA visualization and data labels to show high Pearson value (arc shape) corresponding to better robustness of the network towards noise (Fig. S44). For both networks, we used signal to noise ratios (SNR) of ∞ (no noise-original images), 0.1, 0.3 and 0.5. For both networks, MDA visualizations become more arc-shaped and color distribution becomes more ordered with improvements in SNR. However, at the same SNR level, the MDA visualization of Dense-UNet features is better arc-shaped and color distribution is more ordered than in MDA visualizations of the simple U-Net features. MDA also reveals the robustness of DNNs to noise on the feature space for classification tasks (see Figs. S45 and S46 added below).

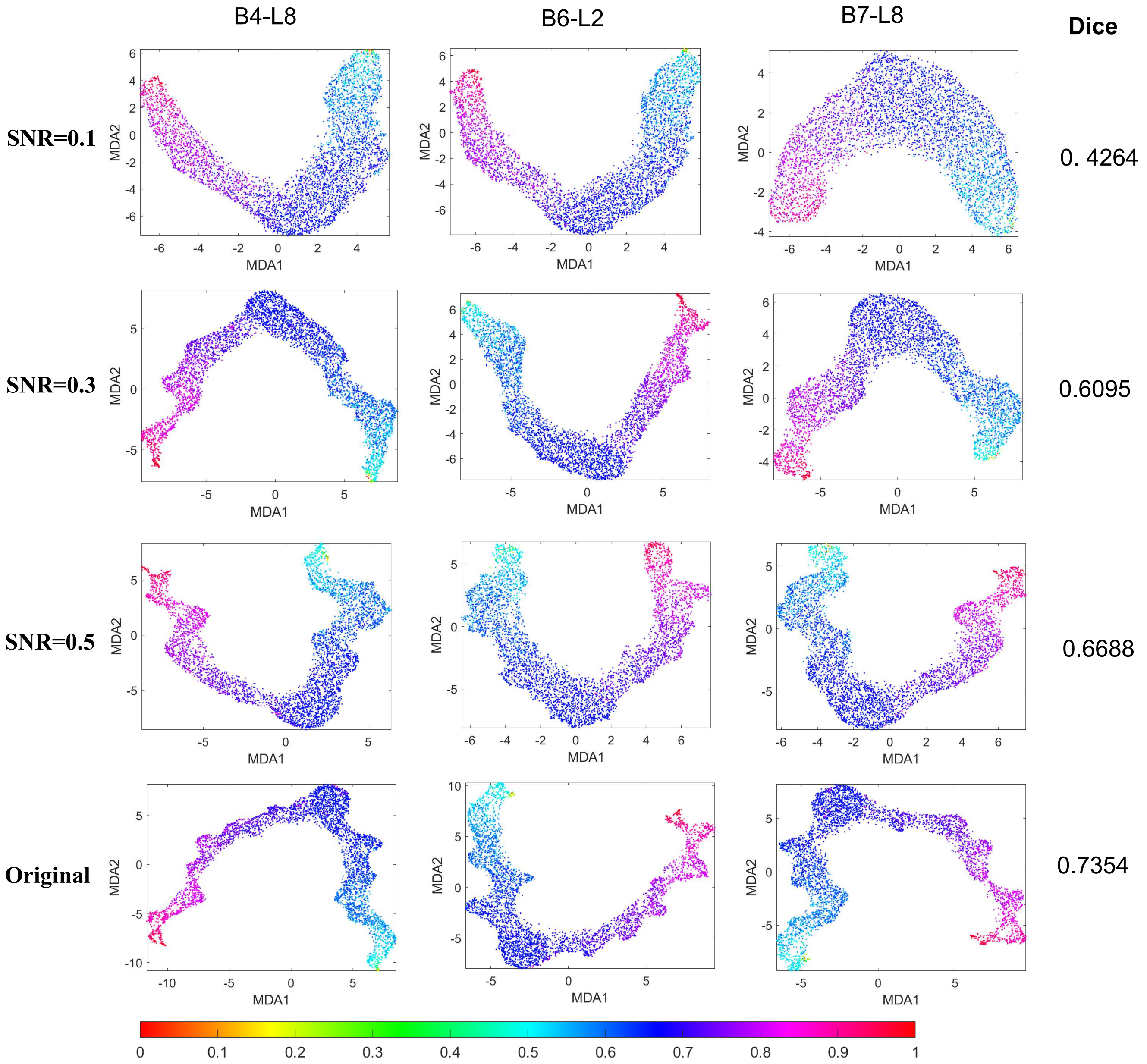

Fig. S42. Visualization of test data features for Dense-UNet image segmentation network (see Fig. 3) with random noise. This experiment involved adding noise to both the training and testing sets and examining the intermediate layers of the network after training. Signal to noise ratios (SNR) of ∞ (no noise-original images), 0.1, 0.3 and 0.5 were used. (Row 1) With SNR=0.1, the MDA visualization shows distorted shapes and the colors are not ordered. (Row 2, 3, 4) With increment of SNR, the shape becomes more arc-shaped and colors are ordered. These results suggest that increased noise negatively affects the DNNs' learning process, reducing the quality of the intermediate layer features. The MDA method effectively shows the extent to which noise impacts the feature space of deep neural networks. Segmentation Dice scores are shown on the right. Source data are provided as a Source Data file.

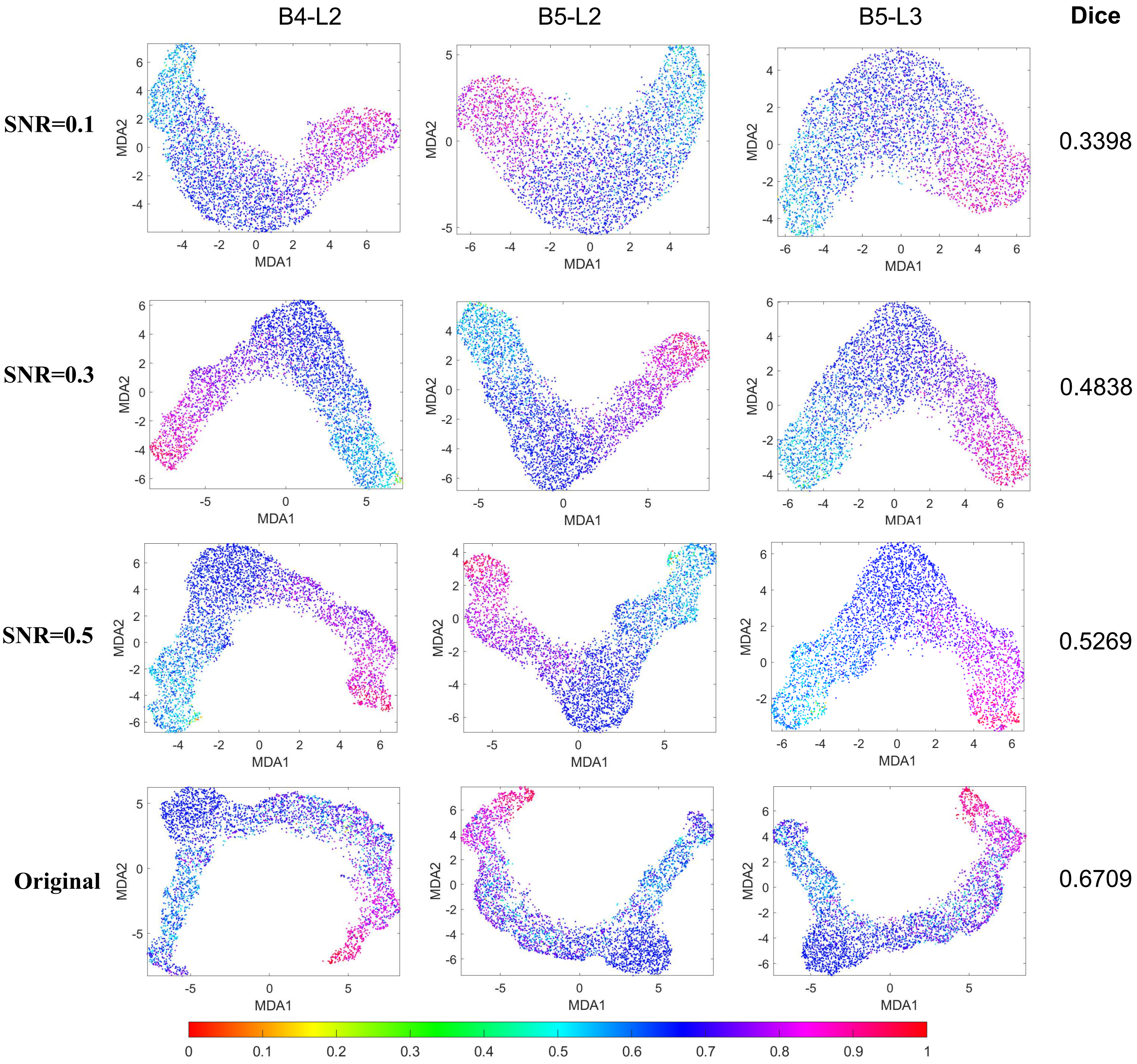

Fig. S43. MDA visualizations of the DNN feature space at different noise levels for simple U-Net. Segmentation Dice scores are shown on the right. Source data are provided as a Source Data file.

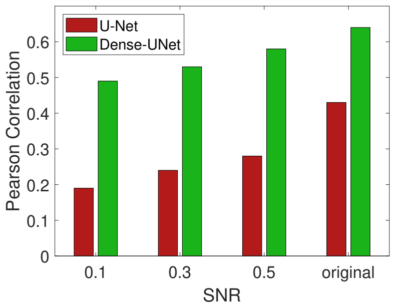

Fig. S44. Pearson correlations between geodesic distances among the HD label data and LD representations from MDA for different noise levels for Dense-UNet and simple U-Net. Source data are provided as a Source Data file.

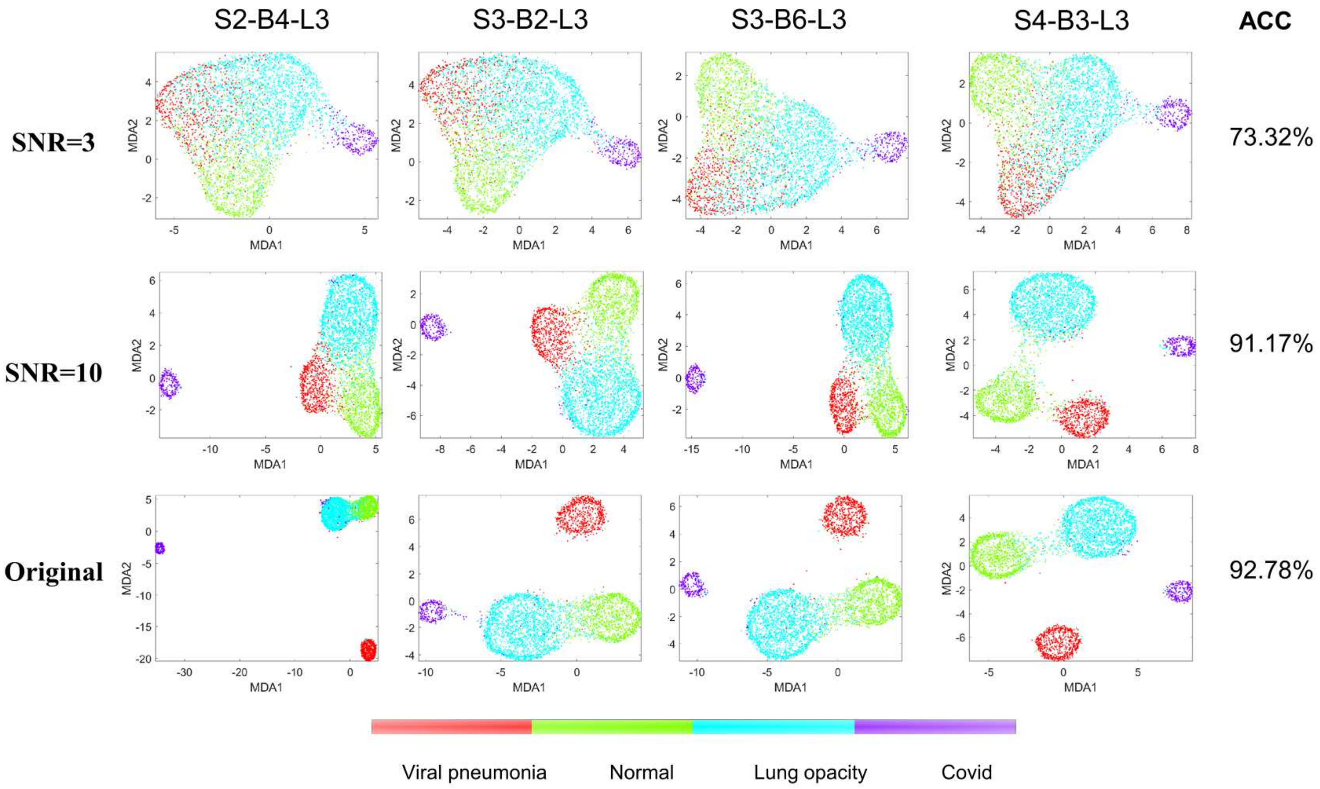

Fig. S45. MDA visualizations of the DNN feature space at different noise levels for ResNet in classification task trained on COVID dataset. Classification accuracies of the network are shown on the right. Source data are provided as a Source Data file.

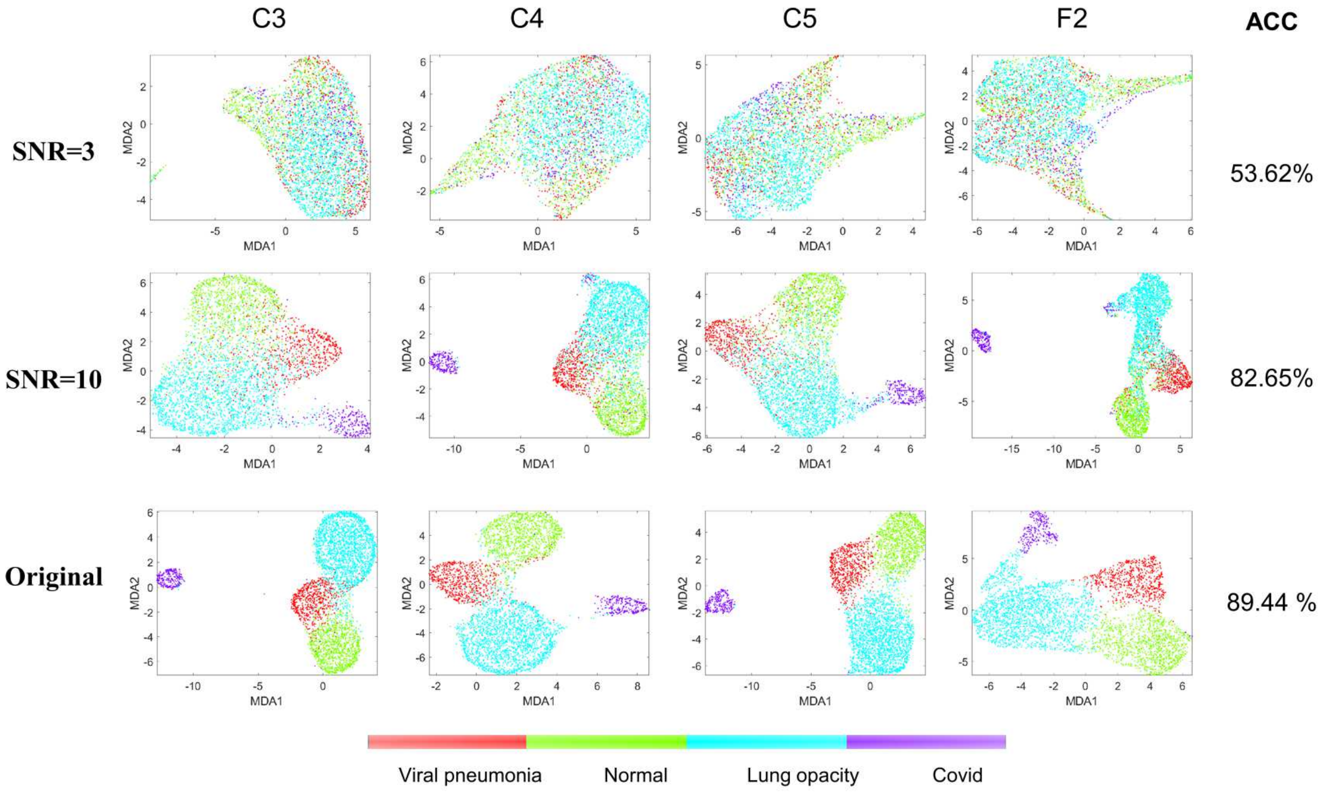

Fig. S46. MDA visualizations of the DNN feature space at different noise levels for AlexNet (15) in classification task trained on COVID dataset. Classification accuracies of the network are shown on the right. Source data are provided as a Source Data file.

14. Supplementary References

1. Diaconis P, Ylvisaker D (1979) Conjugate Priors for Exponential Families. *The Annals of Statistics* 7(2):269–281.
2. McInnes L, Healy J, Melville J (2020) UMAP: Uniform Manifold Approximation and Projection for Dimension Reduction. *arXiv:1802.03426 [cs, stat]*.
3. Becht E, et al. (2019) Dimensionality reduction for visualizing single-cell data using UMAP. *Nature Biotechnology* 37(1):38–44.
4. Sainburg T, McInnes L, Gentner TQ (2021) Parametric UMAP Embeddings for Representation and Semisupervised Learning. *Neural Computation* 33(11):2881–2907.
5. Scott DW (1979) On optimal and data-based histograms. *Biometrika* 66(3):605–610.
6. Redmon J, Divvala S, Girshick R, Farhadi A (2016) You Only Look Once: Unified, Real-Time Object Detection. *arXiv:1506.02640 [cs]*.
7. Deng L (2012) The MNIST Database of Handwritten Digit Images for Machine Learning Research [Best of the Web]. *IEEE Signal Processing Magazine* 29(6):141–142.
8. Tenenbaum JB, de Silva V, Langford JC (2000) A Global Geometric Framework for Nonlinear Dimensionality Reduction. *Science* 290(5500):2319–2323.
9. Papyan V, Han XY, Donoho DL (2020) Prevalence of neural collapse during the terminal phase of deep learning training. *Proceedings of the National Academy of Sciences* 117(40):24652–24663.
10. Kothapalli V (2023) Neural Collapse: A Review on Modelling Principles and Generalization.
11. Liu W, Yu L, Weller A, Schölkopf B (2023) Generalizing and Decoupling Neural Collapse via Hyperspherical Uniformity Gap.
12. Zhou J, et al. (2022) Are All Losses Created Equal: A Neural Collapse Perspective.
13. Zhu Z, et al. (2021) A Geometric Analysis of Neural Collapse with Unconstrained Features.
14. Ronneberger O, Fischer P, Brox T (2015) U-Net: Convolutional Networks for Biomedical Image Segmentation. *arXiv:1505.04597 [cs]*.
15. Krizhevsky A, Sutskever I, Hinton GE (2012) ImageNet Classification with Deep Convolutional Neural Networks in *Advances in Neural Information Processing Systems 25*, eds. Pereira F, Burges CJC, Bottou L, Weinberger KQ. (Curran Associates, Inc.), pp. 1097–1105.
